# Linking formulation composition to pharmacokinetic and pharmacodynamic responses: a predictive framework for sustained-release tablets

**DOI:** 10.1080/10717544.2026.2752652

**Published:** 2026-10-27

**Authors:** Sungmin Song, Meoung Jun Park, Jun Hyung Pyo, Tae Hwan Kim, Beom Soo Shin, Soyoung Shin

**Affiliations:** a School of Pharmacy, Sungkyunkwan University, Suwon, Gyeonggi, Korea; b College of Pharmacy, Daegu Catholic University, Gyeongsan, Gyeongbuk, Korea; c College of Pharmacy, Chung-Ang University, Seoul, Korea; d Department of Global Innovative Drugs, The Graduate School of Chung-Ang University, Seoul, Korea

**Keywords:** In vitro–in vivo correlation, sustained-release tablets, pharmacokinetic–pharmacodynamic modeling, drug delivery, formulation optimization

## Abstract

The development of sustained-release formulations remains largely empirical and often requires iterative in vitro and in vivo studies to achieve desired pharmacokinetic profiles and therapeutic outcomes. In this study, we developed a formulation-driven predictive framework that links formulation composition to pharmacokinetic and pharmacodynamic responses by integrating design of experiments (DoE), in vitro–in vivo correlation (IVIVC), and population pharmacokinetic/pharmacodynamic (PK/PD) modeling. This approach enables the prediction of both plasma concentration–time and pharmacodynamic response–time profiles directly from formulation composition. Using ticagrelor as a model drug, nineteen sustained-release formulations were prepared and characterized in vitro based on a mixture design with three formulation variables. Three formulations spanning fast, medium, and slow dissolution rates were selected for in vivo pharmacokinetic and pharmacodynamic evaluation in beagle dogs. In vitro dissolution kinetics were described using a Michaelis–Menten model, and the maximum dissolution rate (V_max_) was used as a common linking parameter across the DoE, IVIVC, and PK/PD components. A population PK/PD model incorporating time-dependent dissolution and site-dependent absorption processes was established, and a Level A IVIVC was developed by correlating in vitro and in vivo V_max_ values. The framework was validated using both internal and external formulations and successfully predicted pharmacokinetic and pharmacodynamic profiles from formulation composition alone. Prediction errors were within 12.70% for internal validation formulations and within 9.67% for an additionally prepared external validation formulation, meeting FDA criteria for IVIVC predictability. This framework provides a mechanistically grounded strategy for model-informed optimization of sustained-release tablet delivery systems.

## Graphical abstract

**Figure:**
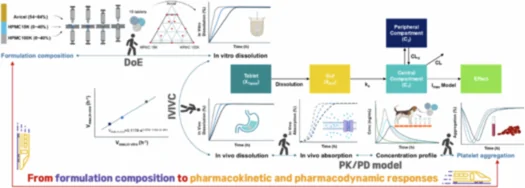

## 1. Introduction

Sustained-release (SR) formulations are designed to improve therapeutic consistency and patient compliance by reducing peak–trough fluctuations. The development of SR formulations is, however, a complex process that requires balancing multiple formulation variables to achieve both desired in vitro release and acceptable in vivo performance. The drug release rate from an SR matrix is governed by complex interactions among its components, including polymers, diluents, and the drug itself (Siepmann and Siepmann 2012). The resulting in vivo performance is further complicated by physiological factors such as gastrointestinal transit, luminal pH, regional differences in permeability, and site-dependent absorption (Mackie et al. 2024). Conventionally, this formulation optimization relies on an iterative cycle of formulation design, in vitro dissolution testing, and in vivo evaluation. Nevertheless, establishing a satisfactory in vitro dissolution profile does not guarantee acceptable in vivo performance, particularly for drugs with complex absorption characteristics. Each new formulation candidate, therefore, typically requires a separate in vivo study to confirm its performance, making the optimization process time-consuming and resource-intensive. This reliance on iterative experimentation remains a key limitation in rational sustained-release formulation development.

In vitro–in vivo correlation (IVIVC) has been widely used to overcome this limitation by establishing a predictive relationship between in vitro dissolution and in vivo drug absorption (FDA US 1997; Kim et al. 2024; Wang et al. 2025). Meanwhile, design of experiments (DoE), particularly the mixture design, offers a systematic approach to relate formulation composition to in vitro properties, including dissolution behavior (Politis et al. 2017). However, IVIVC alone does not relate formulation composition to in vivo performance, and DoE alone addresses only the in vitro stage. These approaches are typically applied in isolation, limiting the ability to translate formulation composition directly into in vivo performance. To date, no established framework has systematically linked formulation composition to therapeutic outcomes within a unified approach.

To address this limitation, the concept of extended IVIVC has been proposed, in which DoE and IVIVC models are incorporated into a population pharmacokinetic model to form a single predictive framework (Lim et al. 2022). The extended IVIVC enables the prediction of in vivo pharmacokinetic profiles directly from formulation composition. Conversely, it can also be used to identify formulation compositions that achieve a desired pharmacokinetic profile. This approach was first demonstrated for baclofen SR tablets (Kim et al. 2019) and subsequently advanced for ketoprofen SR tablets using a mixture design with multiple formulation factors (Lim et al. 2022).

However, these approaches primarily focus on pharmacokinetics and do not extend to pharmacodynamic outcomes, which ultimately determine therapeutic success. In formulation development, the goal is not merely to control systemic exposure but to achieve a desired therapeutic response. If in vivo pharmacodynamic profiles could also be predicted, formulation optimization could be guided directly by the expected pharmacological response. For example, a target intensity or duration of pharmacological effect could be translated back into a specific formulation composition without iterative in vivo testing. Such an approach would enable a more rational and outcome-driven formulation design strategy. Achieving this goal requires integrating a population pharmacokinetic/pharmacodynamic (PK/PD) model into the extended IVIVC framework, which has not previously been pursued.

Ticagrelor, a reversible P2Y12 receptor antagonist, is widely used to prevent atherothrombotic events (Wallentin et al. 2009; Bonaca et al. 2015). It currently requires twice-daily dosing due to its short duration of antiplatelet effect (Butler and Teng 2010), and an SR formulation could enable sustained platelet inhibition. Moreover, its antiplatelet effect is directly related to plasma concentration (Astrand et al. 2019), making it well-suited for pharmacodynamic modeling. At the same time, as a biopharmaceutics classification system (BCS) class IV drug with low solubility and permeability (Teng and Maya 2014), it presents formulation challenges in which both dissolution and absorption may vary with gastrointestinal transit. These characteristics hinder the establishment of reproducible release–absorption relationships across formulations, highlighting the need for an integrated and predictive formulation design framework.

Therefore, the objective of the present study was to develop an integrated extended IVIVC–PK/PD framework that links formulation composition to pharmacokinetics and pharmacodynamic response. The framework integrates DoE, IVIVC, and population PK/PD modeling into a unified end-to-end approach. The proposed framework mechanistically links formulation composition to dissolution, absorption, systemic exposure, and pharmacodynamic response, thereby enabling direct prediction of pharmacokinetic as well as pharmacodynamic responses from formulation design. This approach provides a practical strategy for formulation optimization by reducing reliance on iterative in vitro and in vivo studies. Using ticagrelor as a model drug, nineteen SR formulations were prepared using mixture design, and in vivo pharmacokinetics and antiplatelet effects were evaluated in beagle dogs. The predictive capability of the framework was validated by predicting both the plasma concentration–time and antiplatelet effect–time profiles of a prospectively designed independent formulation solely from formulation composition.

## 2. Materials and methods

### 2.1. Materials

Ticagrelor was obtained from Wuxi Hexia Chemical Company (Jiangsu, China), and ticagrelor-d7, used as the internal standard (IS), was sourced from Sigma-Aldrich (St. Louis, MO). Two grades of hydroxypropyl methylcellulose (HPMC 2208–15,000 cps and HPMC 2208–100,000 cps) were supplied by Shin-Etsu Chemical Co., Ltd (Tokyo, Japan). Microcrystalline cellulose, magnesium stearate, potassium dihydrogen phosphate, sodium hydroxide, hydrochloric acid, and sodium chloride were purchased from Samchun Chemical Co., Ltd (Seoul, Korea). HPLC-grade acetonitrile, ethanol, and water were obtained from J.T. Baker Co. (Philipsburg, NJ).

### 2.2. Animals

Male beagle dogs (*n* = 20, 11–12 months of age, 8.3–12.8 kg body weight) were supplied by Orient Bio Inc. (Seongnam, Korea). The sample size of *n* = 4 per treatment group was based on our prior pharmacokinetic studies of sustained-release formulations in beagle dogs. All in-life procedures were conducted at KNOTUS Co., Ltd. (Incheon, Korea), under controlled environmental conditions (20–26 °C, 30–70% relative humidity, 12-h light/dark cycle). Animals were fed a standard dog diet (Cargil Agri Purina, Seongnam, Korea) once daily, with water provided ad libitum except during the overnight fasting period prior to dosing. A minimum 7-day acclimatization period was provided before the study initiation.

All procedures were approved by the KNOTUS Institutional Animal Care and Use Committee (approval number: KNOTUS IACUC 20-KE-520; approval date: 15 September 2020). The animal facility is licensed by the Korean Ministry of Food and Drug Safety (License No. 405), and the KNOTUS IACUC is licensed by the Animal and Plant Quarantine Agency of Korea (License No. AEC-20120608-0001). All experimental procedures were conducted in compliance with the Korean Animal Protection Act and the NIH Guide for the Care and Use of Laboratory Animals (8th edition). The study is reported in accordance with the ARRIVE 2.0 guidelines (Percie du Sert et al. 2020).

### 2.3. Preparation of sustained-release tablets

#### 2.3.1. Mixture design

SR formulations containing 10 mg of ticagrelor were prepared based on a mixture design in Minitab^®^ software version 18 (Minitab Inc., State College, PA). Two types of HPMC, i.e. HPMC 2208-15,000 cps and HPMC 2208-100,000 cps, were used as the drug release rate modifier, while Avicel^®^ and magnesium stearate were used as a diluent and lubricant, respectively. According to the mixture experimental design, 13 formulations were randomly arranged (Table 1, Figure 1A). The fraction of Avicel (x_1_) in a range from 54.0 to 84.0%, the fraction of HPMC 2208–15,000 cps (x_2_) in a range from 0.0 to 40.0%, and the fraction of HPMC 2208–100,000 cps (x_3_) in a range from 0.0 to 40.0% were independent variables. All of the runs have the fixed amounts of ticagrelor 5% and magnesium stearate 1%. Six additional SR formulations of ticagrelor (V1 ~ V6) were designed to validate the DoE model (Table 1).

**Table 1.** Experimental design for ticagrelor sustained-release (SR) formulations and the observed response factors.

Run order	Fixed factors		Control factors			Response factors	
	Ticagrelor (%)	Mg Stearate (%)	x_1_	x_2_	x_3_	T_80_ (h)	1/V_max, in vitro_ (h)
			Avicel (%)	HPMC 15,000 cps (%)	HPMC 100,000 cps (%)		
1	5	1	84	5	5	1.00	1.27
2	5	1	69	25	0	5.11	6.25
3	5	1	61.5	6.25	26.25	4.53	5.26
4	5	1	54	0	40	11.50	13.70
5	5	1	84	0	10	0.89	1.35
6	5	1	54	40	0	10.44	12.20
7	5	1	54	20	20	8.99	10.20
8	5	1	61.5	26.25	6.25	6.23	7.69
9	5	1	84	10	0	1.39	1.72
10	5	1	69	0	25	3.36	4.35
11	5	1	76.5	11.25	6.25	3.03	3.85
12	5	1	69	12.5	12.5	3.58	4.55
13	5	1	76.5	6.25	11.25	2.75	3.45
V1	5	1	81	6.6	6.4	2.05	2.63
V2	5	1	72	16.9	5.1	3.9	5.13
V3	5	1	64.6	14.5	14.9	3.98	4.99
V4	5	1	63.4	4.8	25.8	3.97	4.89
V5	5	1	55.4	19.2	19.4	8.05	9.86
V6	5	1	58.6	17.4	18	6.00	7.38

#### 2.3.2. Tablet preparation

Ticagrelor SR tablets were manufactured using a wet granulation process. In a polyethylene bag, ticagrelor was blended with Avicel, HPMC 2208–15,000 cps, and HPMC 2208–100,000 cps for 10 min. A 60% ethanol solution was then added as the binder, and the resulting wet mass was kneaded. The wet mass was screened through a 20-mesh sieve, and the obtained granules were oven-dried at 50 °C for 1 h. The dried granules were re-screened through a 20-mesh sieve, blended with magnesium stearate for 5 min, and weighed. Tablet compression was carried out at 1 ton on a hydraulic tablet press (Carver, Inc., Wabash, IN) fitted with a 7.0 mm round flat-faced punch.

### 2.4. In vitro dissolution

In vitro dissolution testing of ticagrelor was carried out using the USP paddle apparatus on a Distek Dissolution System 2500 equipped with an Evolution Dissolution Sampler 4300 (Distek, North Brunswick, NJ). Each dissolution vessel contained 900 mL of 0.1 *N* HCl (pH 1.2) supplemented with 0.1% Tween 80, maintained at 37 ± 0.5 °C with a paddle speed of 75 rpm. At 0.17, 0.5, 1, 1.5, 2, 3, 4, 6, 8, 12, 16, 20, 24, and 32 h, 2 mL aliquots were withdrawn by the autosampler, and an equal volume of fresh medium was replenished to maintain sink conditions. Aliquots were subsequently filtered through a 45 μm polyethylene syringe filter (Distek, North Brunswick, NJ) prior to HPLC analysis.

Ticagrelor in the dissolution samples was quantified by HPLC using a Waters Alliance 2695 separation module connected to a Waters 2487 dual-wavelength absorbance detector (Waters, Milford, MA). Chromatographic separation was achieved on a Zorbax 300-SB C18 column (250 × 4.6 mm i.d., 5 μm; Agilent Technologies, Santa Clara, CA) with UV detection at 223.2 nm. The mobile phase was a 45:55 (v/v) mixture of acetonitrile and 20 mM sodium phosphate buffer (pH adjusted to 3.0 with phosphoric acid).

### 2.5. In vivo pharmacokinetics and pharmacodynamics

#### 2.5.1. Animal studies

Three formulations spanning fast (V1), medium (V3), and slow (V5) dissolution rates were selected for in vivo pharmacokinetic and pharmacodynamic evaluation, in accordance with the FDA guidance for IVIVC development, which recommends three or more formulations with different release rates (FDA US 1997). The in vivo study was conducted between September and December 2020. Beagle dogs were randomly allocated to four treatment groups using a computer-generated random sequence: intravenous (IV) injection (*n* = 4), or oral administration of V1, V3, or V5 (*n* = 4 each). All animals were fasted overnight (≥12 h) prior to dosing, with water provided ad libitum. For IV administration, a 3 mg/mL ticagrelor solution was freshly prepared on the day of dosing by dissolving ticagrelor powder in a vehicle composed of PEG and sterile normal saline (40:60, v/v). For oral administration, each tablet was administered with 10 mL of water. Blood samples for PK and PD analyzes were drawn from the cephalic vein by manual restraint without anesthesia or sedation, and collected into heparinized tubes (5 IU/mL) at pre-dose (0 h) and 0.25, 0.5, 0.75, 1, 1.5, 2, 2.5, 3, 4, 5, 6, 8, 12, and 24 h post-dose. Plasma was separated by centrifugation (4,000 × g, 4 °C, 10 min) and stored at –70 °C until analysis. Pharmacokinetic analysis was performed on all four animals per treatment group (*n* = 4). Because whole blood impedance aggregometry requires immediate processing of fresh blood within a limited time window, pharmacodynamic analysis was performed on three of the four animals per oral administration group (*n* = 3); the IV group was not subjected to pharmacodynamic analysis. Bioanalytical (LC-MS/MS) and pharmacodynamic (whole blood impedance aggregometry) assays were performed using coded samples to minimize operator bias. After completion of the study, animals were returned to the KNOTUS facility for use in other approved studies.

#### 2.5.2. Liquid chromatography-tandem mass spectrometry (LC-MS/MS)

Plasma ticagrelor concentrations in beagle dogs were measured by an LC-MS/MS assay, with ticagrelor-d7 serving as the internal standard (IS). The instrumentation consisted of an Agilent 1260 HPLC system interfaced with an Agilent 6490 triple-quadrupole mass spectrometer (Agilent Technologies, Santa Clara, CA). Chromatographic separation was achieved on an EclipsePlus C18 RRHD column (50 × 2.1 mm i.d., 1.8 μm; Agilent Technologies, Santa Clara, CA) under isocratic elution with acetonitrile/0.1% aqueous formic acid (55:45, v/v) as the mobile phase. Operating conditions were as follows: flow rate, 0.3 mL min^−1^; column oven temperature, 40 °C; total run time, 6 min. Ionization was carried out via electrospray in positive ion mode, and detection was performed in multiple reaction monitoring (MRM) mode with a dwell time of 200 ms. The precursor-to-product ion transitions monitored were m/z 523.2 → 495.2 for ticagrelor and m/z 530.2 → 370.2 for ticagrelor-d7.

Full method validation was conducted using matrix-matched quality control samples in accordance with FDA bioanalytical guidance (FDA U 2018). The lower limit of quantification (LLOQ) for ticagrelor in beagle dog plasma was 0.5 ng mL^−1^. Intra-day accuracy ranged from 93.86 to 105.81% (*n* = 5), and inter-day accuracy from 100.24 to 105.37% (*n* = 5). Intra-day and inter-day precision were within 7.45% and 9.19%, respectively.

#### 2.5.3. Whole blood impedance aggregometry

Platelet function was assessed by whole blood impedance aggregometry on a Chrono-Log Model 591 aggregometer (Chrono-Log, Havertown, PA) (Kurata et al. 1995; Shin et al. 2015). Venous blood was drawn into heparin-containing syringes to give a final heparin concentration of 5 IU/mL. Samples were held at room temperature (25 °C) on a roller mixer with gentle agitation prior to analysis.

For each measurement, whole blood was combined with an equal volume of 0.9% NaCl. The resulting 1:1 mixture (500 μL of each) was loaded into a siliconized plastic cuvette pre-fitted with a magnetic stir bar and positioned in the aggregometer. An impedance electrode was then inserted, and the sample was stirred at 1,200 rpm and 37 °C until a stable baseline impedance was reached. Aggregation was induced by addition of adenosine diphosphate (ADP) to a final concentration of 10 μM, and the impedance signal was monitored for 10 min. Aggregation magnitude was quantified as the area under the impedance–time curve.

### 2.6. Model development

The modeling workflow was designed to predict pharmacokinetic and pharmacodynamic profiles directly from formulation composition.

#### 2.6.1. DoE model

The time to 80% dissolution (T_80_) was calculated from the in vitro dissolution profiles of the thirteen DoE formulations. In parallel, the dissolution kinetics were described using a simple kinetic model in which the dissolution rate served as the shared linking parameter of the framework (Kim et al. 2019; Lim et al. 2022). Michaelis-Menten and first-order kinetics were considered as candidates, and the Michaelis-Menten model provided a better fit for the in vitro dissolution profiles of the thirteen formulations. The Michaelis–Menten kinetic equation was characterized by two parameters: the maximum dissolution rate (V_max, in vitro_) and the amount of undissolved drug corresponding to half-maximum rate (AM_50_). The rate of change in undissolved drug amount in the dissolution medium (X_tablet, in vitro_) was expressed as:

(1) $$\frac{dX_{\mathrm{tablet},\;\mathrm{invitro}}}{\mathrm{dt}}=-\mathrm{Dose}\;∙\;V_{\max,\;\mathrm{invitro}}\;∙\;\frac{X_{\mathrm{tablet},\mathrm{invitro}}}{{\mathrm{Dose}\;∙\;\mathrm{AM}}_{50}+\;X_{\mathrm{tablet},\mathrm{invitro}}}$$

Formulation-specific V_max, in vitro_ values and a shared AM_50_ were estimated by nonlinear regression in Monolix 2018 R (Lixoft, Antony, France). The reciprocal value (1/V_max, in vitro_) served as the response variable in the subsequent DoE analysis and as the linking parameter for IVIVC development.

Response surface modeling relating the three formulation factors (x_1_, x_2_, x_3_) to T_80_ and 1/V_max, in vitro_ was conducted in Minitab (Minitab Inc., State College, PA), and the best-fit polynomial equation was selected for each response. Model adequacy was assessed using R^2^, adjusted R^2^, and predicted R^2^ statistics, and contour plots were constructed to visualize the response surfaces.

To evaluate the predictive performance of the DoE model, six additional SR formulations (V1–V6, Table 1) were prepared as an independent validation batch, with compositions selected outside the DoE training set. For each validation formulation, observed T_80_ and 1/V_max, in vitro_ were compared with the corresponding model predictions, and prediction error (PE%) was computed as:

(2) $$\mathrm{PE}(\%)=\frac{\left|\mathrm{Predicted}-\mathrm{Observed}\right|}{\mathrm{Observed}}\times 100\%$$

#### 2.6.2. Population PK/PD model

In the population PK/PD model, in vivo dissolution of ticagrelor from the SR tablet was assumed to follow Michaelis–Menten kinetics, with formulation-specific V_max, in vivo,_ and shared AM_50_. The change in the amount of undissolved ticagrelor in the gastrointestinal tract (X_tablet, in vivo_) over time was expressed as:

(3) $$\frac{dX_{\mathrm{tablet},\;\mathrm{invivo}}}{\mathrm{dt}}=-\mathrm{Dose}\;∙\;F_{\mathrm{abs}}\;∙\;V_{\max,\;\mathrm{invivo}}\;∙\;\frac{X_{\mathrm{tablet},\;\mathrm{invivo}}}{{\mathrm{Dose}∙F_{\mathrm{abs}}\;∙\;\mathrm{AM}}_{50}+\;X_{\mathrm{tablet},\mathrm{invivo}}}$$

Here, V_max, in vivo_ denotes the maximum in vivo dissolution rate, and F_abs_ represents the oral bioavailability of ticagrelor. To capture potential delays in tablet dissolution in vivo, a lag time (T_lag_) was incorporated; dX_tablet, in vivo_/dt was constrained to 0 prior to T_lag_.

Dissolved ticagrelor (X_gut_) subsequently entered the systemic circulation from the gut compartment, governed by the absorption rate constant k_a_.

(4) $$\frac{dX_{\mathrm{gut}}}{\mathrm{dt}}=\mathrm{Dose}\;∙\;F_{\mathrm{abs}}\;∙\;V_{\max,\;\mathrm{invivo}}\;∙\;\frac{X_{\mathrm{tablet},\;\mathrm{invivo}}}{{\mathrm{Dose}∙F_{\mathrm{abs}}\;∙\;\mathrm{AM}}_{50}+\;X_{\mathrm{tablet},\;\mathrm{invivo}}}-k_{a}\;∙\;X_{\mathrm{gut}}\;$$

To capture the site-dependent absorption behavior of ticagrelor, k_a_ was modeled as time-dependent using a sigmoidal term.

(5) $$k_{a}=k_{a}^{\prime }∙\left(1+{\mathrm{Abs}}_{\max}∙\frac{{\mathrm{Time}}^{\gamma }}{T_{\mathrm{get}}^{\gamma }+{\mathrm{Time}}^{\gamma }}\right)\;$$

In Equation 5, k_a_’represents the basal absorption rate constant, Abs_max_ denotes the relative increase in k_a_ at the absorption site, Time is the elapsed time post-dose, and T_get_ is the time at which absorption enhancement reaches its midpoint. k_a_ therefore transitions sigmoidally from k_a_’ to k_a_’∙(1+Abs_max_), with the midpoint occurring at Time = T_get_ and the Hill coefficient *γ* controlling the steepness of the transition. This time-dependent parameterization is a semi-mechanistic representation of gastrointestinal transit and site-dependent absorption, using time as a surrogate for anatomical position along the gastrointestinal tract.

Systemic disposition of ticagrelor was modeled using a two-compartment structure (central and peripheral compartments), assuming linear kinetics. Ticagrelor (X_1_) underwent distribution between the central compartment and the peripheral compartment (X_2_), with elimination occurring from the central compartment.

(6) $$\frac{dX_{1}}{\mathrm{dt}}=k_{a}∙X_{\mathrm{gut}}-{\mathrm{CL}}_{d}∙C_{1}+{\mathrm{CL}}_{d}∙C_{2}-\mathrm{CL}∙C_{1}$$

(7) $$\frac{dX_{2}}{\mathrm{dt}}={\mathrm{CL}}_{d}∙C_{1}-{\mathrm{CL}}_{d}∙C_{2}$$

C_1_ and C_2_ represent the central and peripheral concentrations of ticagrelor, respectively. CL_d_ denotes the distribution clearance between compartments, and CL is the systemic clearance.

A direct inhibition model was used to characterize the PD of ticagrelor, assuming an instantaneous concentration-response relationship consistent with the reversible P2Y12 receptor binding of ticagrelor. The relative platelet aggregation (%) was described by a Hill-type inhibition expression:

(8) $$\mathrm{Aggregation}\left(\%\right)=100\times {(1-I}_{\max}∙\;\frac{C_{1}^{n}}{{\mathrm{IC}}_{50}^{n}+C_{1}^{n}})\;$$

In Equation 8, I_max_represents the maximum fractional inhibition, IC_50_ denotes the ticagrelor plasma concentration producing half-maximal inhibition, C_1_ is the central compartment concentration, and *n* is the Hill coefficient. At baseline (C_1_ = 0), the aggregation equals 100%, and at sufficiently high concentrations, the aggregation approaches 100 × (1 − I_max_).

Population PK parameters (including V_max, in vivo_) and PD parameters were estimated simultaneously by fitting ticagrelor plasma concentration–time data and corresponding platelet aggregation measurements to the PK/PD model in S-ADAPT®. A nonparametric bootstrap analysis was performed by resampling individuals with replacement to generate 200 datasets, each of which was refitted with the final model. Runs that did not reach the specified number of iterations or yielded estimates deviating more than tenfold from the original values were excluded, and the median and 95% confidence intervals of the remaining estimates were compared with the original estimates. In addition, a local sensitivity analysis was performed by perturbing each PK and PD parameter individually by ±25% and ±50% from its final estimate, with all other parameters fixed, and C_max_, AUC, E_max_, and AUEC simulated for the V1, V3, and V5 formulations were expressed as a percentage of the unperturbed simulation.

#### 2.6.3. Extended IVIVC-PK/PD model

An IVIVC for ticagrelor SR tablets was developed by regression-based correlation of the in vitro (V_max, in vitro_) and in vivo (V_max, in vivo_) dissolution parameters. The resulting regression equation was integrated into the population PK/PD model, enabling translation of in vitro dissolution into the corresponding in vivo profile.

The extended IVIVC–PK/PD model was subsequently constructed by adding the DoE equation to this PK/PD framework. The DoE equation linked formulation composition to V_max, in vitro_. Through the IVIVC equation, V_max_ served as the shared connecting parameter across formulation composition, in vitro dissolution, in vivo dissolution, plasma exposure, and pharmacodynamic response within a single integrated framework.

For model validation, 1,000 individual plasma concentration–time and pharmacodynamic effect–time profiles were generated by Monte Carlo simulation in Berkeley Madonna (version 8.3.18) using the finalized extended IVIVC–PK/PD model. C_max_ and AUC were derived from each predicted PK profile, while E_max_ and AUEC were computed from each predicted PD profile. The predicted values were compared with observed data, and predictive performance was assessed using prediction error (PE%).

### 2.7. Model application

The finalized extended IVIVC–PK/PD model was applied to predict in vivo PK (plasma concentration–time) and PD (antiplatelet effect–time) profiles directly from formulation composition. A ticagrelor SR formulation composition (V6) was randomly selected from the design space, and its in vivo PK and PD profiles were generated by Monte Carlo simulation (*n* = 1,000) using the model. In parallel, V6 tablets were manufactured by wet granulation and orally administered to beagle dogs (*n* = 4) to obtain the corresponding observed data. The V6 in vivo study followed the same animal procedures, randomization, sample handling, and ethical approval (KNOTUS IACUC 20-KE-520) as described in Section 2.5.1. The observed profiles were subsequently compared with the model-predicted profiles, and prediction errors (PE%) were determined for C_max_, AUC, E_max_, and AUEC.

A formulation sensitivity analysis was performed to evaluate the effect of formulation composition on PK and PD responses with the extended IVIVC–PK/PD model. Because the mixture design constrains the sum of the three components, each component was varied one at a time around the V3 center point while keeping the ratio of the other two constant (Figure 2A): HPMC 15,000 (x_2_) and HPMC 100,000 (x_3_) by ± 25% and ± 50%, and Avicel (x_1_) by −15%, −5%, +10%, and +25% within its feasible range.

### 2.8. Statistical analysis

Pharmacokinetic and pharmacodynamic parameters are presented as mean ± standard deviation (SD). Between-group comparisons were performed by one-way analysis of variance (ANOVA) followed by the least significant difference (LSD) post-hoc test, with a *P* value < 0.05 considered statistically significant. All statistical analyzes were conducted using IBM SPSS Statistics (IBM Corp., Armonk, NY).

## 3. Results

### 3.1. Formulation-dissolution relationship

#### 3.1.1. Model development

A mixture design comprising thirteen ticagrelor SR tablet formulations was implemented to quantify how formulation composition influences in vitro dissolution behavior. Table 1 lists the compositions of the thirteen formulations, with Avicel, HPMC 15,000 cps, and HPMC 100,000 cps fractions assigned as control factors (x_1_, x_2_, and x_3_, respectively), alongside their measured dissolution descriptors (T_80_ and 1/V_max, in vitro_). Figure 1 depicts the simplex design space (A) and the corresponding in vitro dissolution profiles overlaid with model predictions (B).

**Figure 1.**
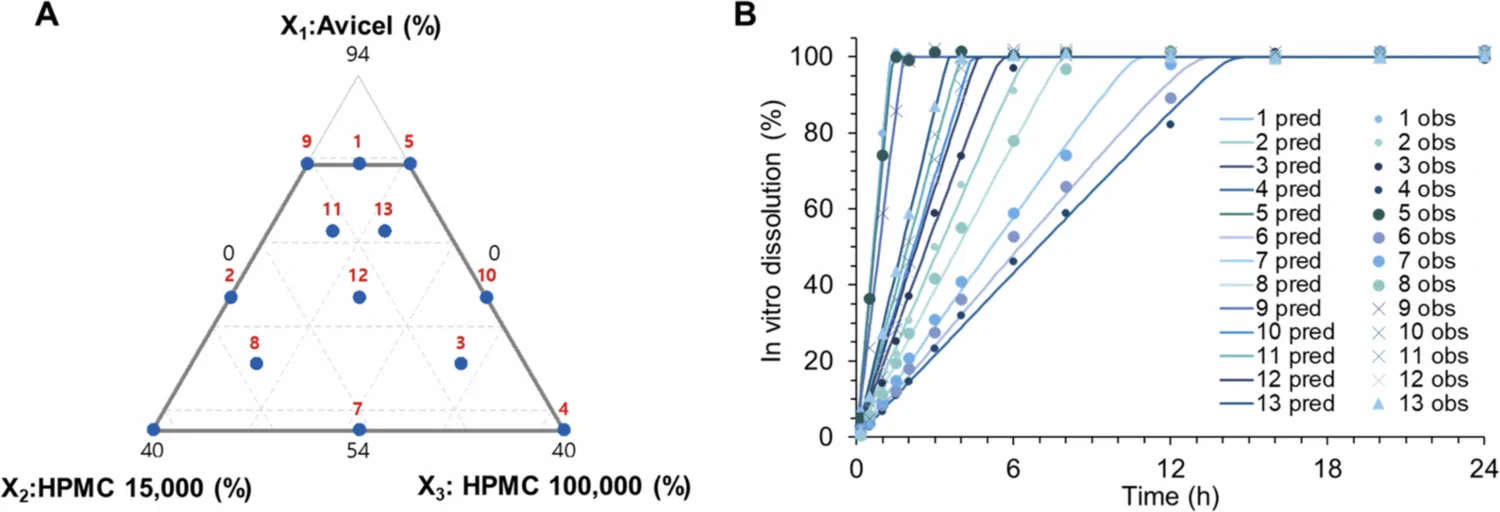
Mixture-design layout of the thirteen ticagrelor sustained-release (SR) tablet compositions (A) and the corresponding in vitro dissolution profiles (B). Observed dissolution data are shown as symbols, with model-fitted profiles overlaid as lines.

Dissolution behavior differed markedly across the thirteen formulations and was well captured by Michaelis–Menten kinetics (Figure 1B). Depending on formulation composition, T_80_ spanned 0.89 to 11.50 h, and the model-derived 1/V_max, in vitro_, covered 1.27 to 13.70 h (Table 1).

The fitted polynomial equations in coded terms linking the three control factors (x_1_, x_2_, x_3_) to T_80_ and 1/V_max, in vitro_ are presented below:

(9) $$T_{80}=-0.07409x_{1}+2.6158x_{2}+3.96975x_{3}-0.04637x_{1}x_{2}-0.07281x_{1}x_{3}-0.00087x_{1}x_{2}x_{3}\;-0.00033\;x_{1}x_{2}(x_{1}{-x}_{2})+0.00046\;x_{1}x_{3}(x_{1}{-x}_{3})-0.00015\;x_{2}x_{3}(x_{2}-x_{3})$$

(10) $${1/V}_{\max\;,\mathrm{invitro}}=-0.08804x_{1}+2.87694x_{2}+4.60076x_{3}-0.05061x_{1}x_{2}-0.08425x_{1}x_{3}\;-0.00102x_{1}x_{2}x_{3}+0.00037\;x_{1}x_{2}(x_{1}-x_{2})+0.00054\;x_{1}x_{3}(x_{1}-x_{3})\;+0.00025x_{2}x_{3}(x_{2}-x_{3})\;$$

The response surfaces of T_80_ and 1/V_max, in vitro_ across the formulation space, are visualized in Supplementary Figure S1. ANOVA outputs for both responses (Supplementary Table S1) returned *P* values below 0.05 for the model terms. F statistics exceeded the critical thresholds and overall *P* values were below the significance level (*α* = 0.05), confirming that both regression models adequately captured the response variability. Goodness-of-fit metrics were R^2^ = 99.94%, adjusted R^2^ = 99.83%, and predicted R^2^ = 97.10% for T_80_, and R^2^ = 99.89%, adjusted R^2^ = 99.66%, and predicted R^2^ = 93.35% for 1/V_max, in vitro_. These high coefficients of determination, together with the statistical significance, confirm the robustness of the established DoE models.

#### 3.1.2. Model validation

Model performance was evaluated by comparing predicted and experimentally measured dissolution behavior for six formulations that fell outside the training set. Six validation compositions (V1–V6), distinct from those used in model training, were chosen from the design space (Figure 2A). Their compositions are summarized in Table 1. Figure 2B overlays the experimentally measured dissolution profiles of these six formulations with the corresponding model predictions.

**Figure 2.**
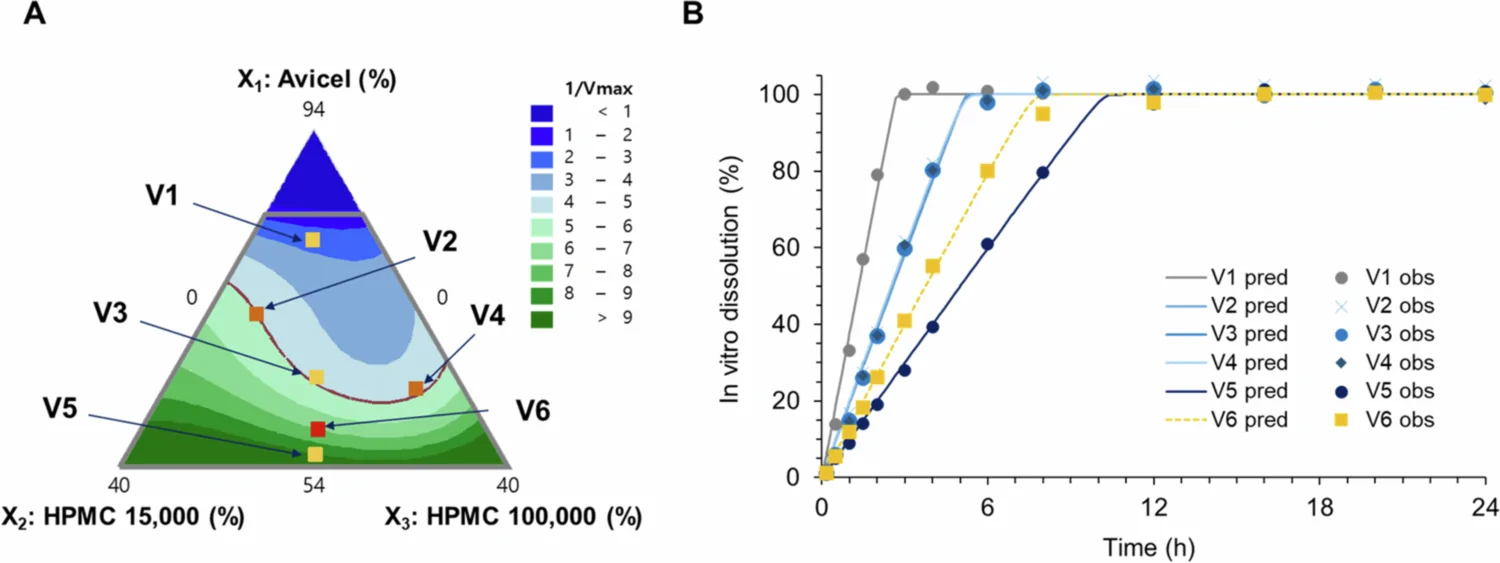
Compositions of the six ticagrelor sustained-release (SR) validation tablets (A) and the overlay of their measured versus model-predicted in vitro dissolution profiles (B). Symbols denote observed dissolution data, and lines indicate model-fitted profiles.

Based on the contour plot (Figure 2A), four compositions (V1, V3, V5, and V6) targeted distinct dissolution rates, whereas three additional compositions (V2, V3, and V4) were designed to produce comparable dissolution profiles. The observed dissolution behavior matched these design intents: V1 released fastest, V5 released slowest, and V2, V3, and V4 produced essentially overlapping profiles (Figure 2B).

Using Equations (9) and (10), the in vitro dissolution descriptors (T_80_ and 1/V_max, in vitro_) for the six validation formulations were predicted from their compositions alone. Comparison with experimentally determined values (Table 2) yielded maximum PE% of 2.76% (mean 1.18%) for T_80_ and 7.08% (mean 3.79%) for 1/V_max, in vitro_. These small prediction errors demonstrate that the DoE model accurately translates formulation composition into in vitro dissolution behavior.

**Table 2.** DoE model validation: comparison of predicted versus observed in vitro dissolution parameters for the ticagrelor SR validation tablets.

Run	T_80_ (h)			1/V_max_ (h)		
	Predicted	Observed	PE (%)	Predicted	Observed	PE (%)
V1	2.01	2.05	1.89	2.55	2.63	3.17
V2	4.01	3.90	2.76	5.03	5.13	1.91
V3	4.00	3.98	0.65	4.85	4.99	2.79
V4	4.00	3.97	0.84	4.77	4.89	2.46
V5	7.98	8.05	0.86	9.16	9.86	7.08
V6	6.00	6.00	0.09	6.98	7.38	5.35

### 3.2. Translation to pharmacokinetics and pharmacodynamics

#### 3.2.1. In vivo PK/PD profiles

Three formulations spanning the in vitro dissolution range—V1 (fast), V3 (medium), and V5 (slow)—were taken forward for the development of the extended IVIVC–PK/PD model. Figure 3 displays the in vivo profiles obtained after oral dosing of V1, V3, and V5 to beagle dogs: plasma concentration–time curves (A) and platelet aggregation–time courses (B). The plasma concentration–time profile after intravenous injection is overlaid for comparison in Figure 3A. Non-compartmental pharmacokinetic parameters of ticagrelor are listed in Table 3.

**Figure 3.**
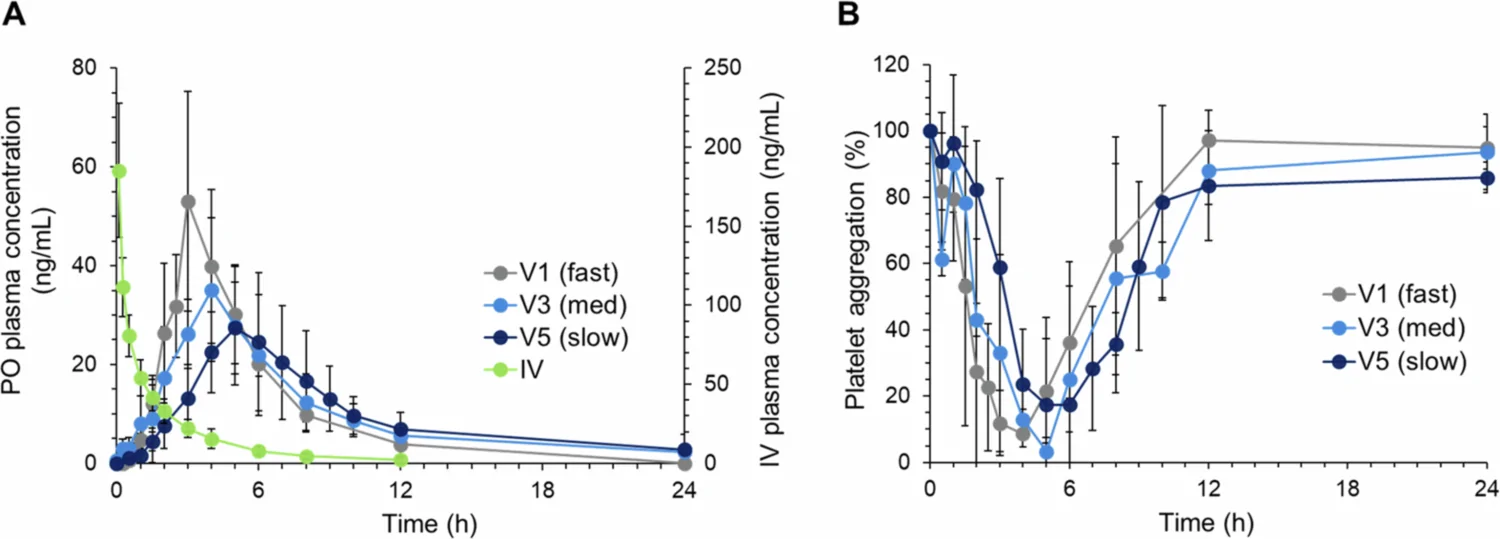
Plasma concentration-time (A) and platelet aggregation-time (B) profiles of ticagrelor following oral administration of the validation formulations (V1, V3, and V5; 10 mg/dog) and intravenous injection (IV; 3 mg/dog) in beagle dogs. Data are presented as mean ± SD (PK: *n* = 4 per group; PD: *n* = 3 per oral administration group).

**Table 3.** Non-compartmental pharmacokinetic parameters of ticagrelor following intravenous injection (IV) or oral administration of SR tablets in beagle dogs (mean ± SD).

Parameter	IV (*n* = 4)	V1 (fast, *n* = 4)	V3 (medium, *n* = 4)	V5 (slow, *n* = 4)
Dose (mg)	3	10	10	10
t_1/2_ (h)	2.37 ± 0.95	2.44 ± 1.19	7.23 ± 1.71*, †	6.22 ± 3.52*^,^ ^†^
T_max_ (h)	-	3.00 ± 0.82	4.00 ± 0.00	5.00 ± 0.82^†^
C_0_ or C_max_ (ng/mL)	239.08 ± 64.93	56.26 ± 18.45	35.12 ± 14.54	29.67 ± 8.52^†^
AUC_last_ (ng·h/mL)	233.17 ± 57.24	208.13 ± 27.41	240.31 ± 93.03	206.25 ± 116.68
AUC_inf_ (ng·h/mL)	243.00 ± 61.99	227.54 ± 22.12	266.79 ± 106.94	243.09 ± 130.41
V_z_ (L)	41.69 ± 8.19	-	-	-
CL (mL/min)	215.27 ± 50.26	-	-	-
Bioavailability (%)	-	26.77 ± 3.53	30.91 ± 11.97	26.53 ± 15.01

Note: -, not applicable;

^*^ , *p* < 0.05 compared with IV;

^†^ , *p* < 0.05 compared with V1.

Following intravenous injection, plasma concentrations declined in a multiexponential manner. After oral administration of SR formulations, the plasma concentration–time profiles showed distinct patterns depending on the dissolution rate (Figure 3A). The apparent half-life of V1 (fast), the fast-releasing formulation, was comparable to that of IV. However, as the dissolution rate decreased, the apparent half-lives of V3 (medium) and V5 (slow) were significantly prolonged compared to IV and V1 (*p* < 0.05). Similarly, T_max_ increased, and C_max_ decreased with slower dissolution rates, with significant differences observed between V1 and V5 (*p* < 0.05). In contrast, AUC values were comparable among the three SR formulations, resulting in similar bioavailability (~27–31%).

Table 4 summarizes the pharmacodynamic parameters of ticagrelor following oral administration of SR tablets. Ticagrelor is a P2Y12 receptor antagonist that inhibits ADP-induced platelet aggregation. As shown in Figure 3B, platelet aggregation decreased rapidly as plasma concentrations increased, reaching maximum inhibition at 3–5 h post-dose. As plasma concentrations declined, the inhibitory effect diminished, and platelet aggregation gradually recovered toward baseline. The average time to maximum effect (T_max, E_) increased with slower dissolution rates (3.33, 4.33, and 5.33 h for V1, V3, and V5, respectively), consistent with the trend observed for T_max_ in the pharmacokinetic profiles. There were no significant differences in E_max_ (maximum antiplatelet effect) and AUEC among the three formulations, indicating that all SR formulations achieved comparable maximum inhibition and overall pharmacodynamic effects regardless of the dissolution rate.

**Table 4.** Pharmacodynamic parameters of ticagrelor following oral administration of SR tablets in beagle dogs (mean ± SD).

Parameter	V1 (fast, *n* = 3)	V3 (medium, *n* = 3)	V5 (slow, *n* = 3)
E_max_ (%)	96.71 ± 1.67	97.37 ± 4.56	90.58 ± 12.59
T_max, E_ (h)	3.33 ± 1.53	4.33 ± 1.15	5.33 ± 0.58
AUEC (%·h)	630.09 ± 46.04	740.57 ± 14.16	722.34 ± 174.54

Note: E_max_, maximum antiplatelet effect, calculated as 100 − minimum aggregation (%); T_max, E_, time to maximum antiplatelet effect; AUEC, area under the effect-time curve.

#### 3.2.2. Population PK/PD model

To estimate the in vivo dissolution of ticagrelor SR tablets under physiological conditions, a population PK/PD model was developed to simultaneously describe the plasma concentration–time profiles and the antiplatelet effect of V1 (fast), V3 (medium), and V5 (slow). The multiexponential decline observed in the plasma concentration–time profiles indicated the need for a two-compartment disposition model. Figure 4 shows the relationship between the observed platelet aggregation and the plasma concentration of ticagrelor across all three SR formulations. The effect declined sigmoidally with increasing plasma concentration, consistent with a direct inhibitory relationship. This pattern supported the use of a direct inhibition model with a Hill expression to describe the antiplatelet effect of ticagrelor.

**Figure 4.**
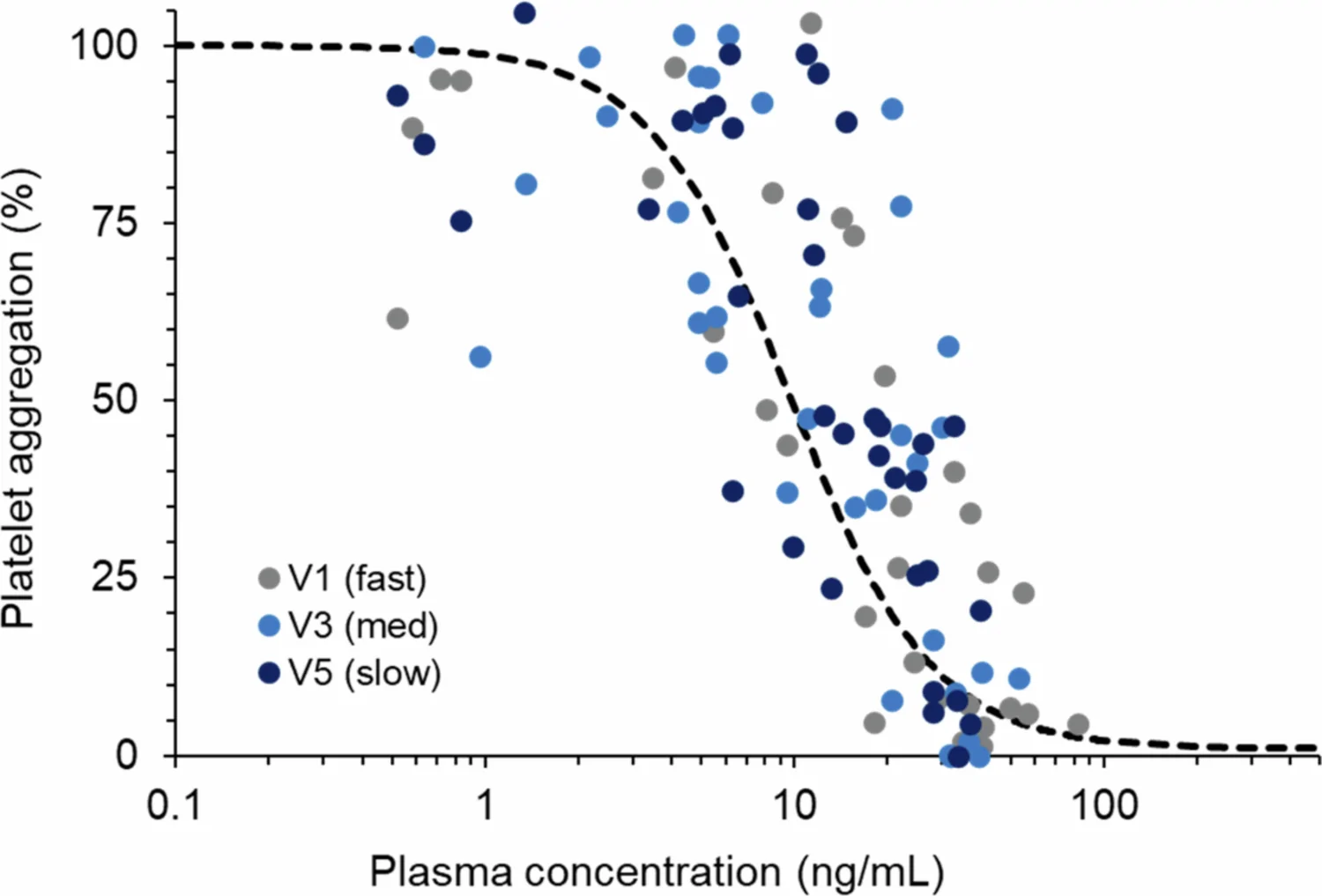
Relationship between plasma ticagrelor concentration and observed platelet aggregation following oral administration of V1 (fast), V3 (medium), and V5 (slow) in beagle dogs. The dotted line represents the fitted curve based on the direct inhibition model.

Based on these observations, the population PK/PD model was constructed as illustrated in Figure 5. The systemic disposition was described by a two-compartment model consisting of a central and a peripheral compartment. The dissolution of ticagrelor from the SR tablet was described by Michaelis–Menten-type dissolution kinetics, and the dissolved drug was subsequently absorbed from the gut compartment into the systemic circulation. The final model incorporated site-specific absorption of ticagrelor through a time-dependent absorption rate constant (k_a_), which increased in a sigmoidal fashion over time. The utility of this time-dependent factor in modulating in vivo dissolution has been demonstrated in previous studies (Kim et al. 2019; Shin et al. 2020). The antiplatelet effect was linked to the plasma concentration using a direct inhibition model as described above.

**Figure 5.**
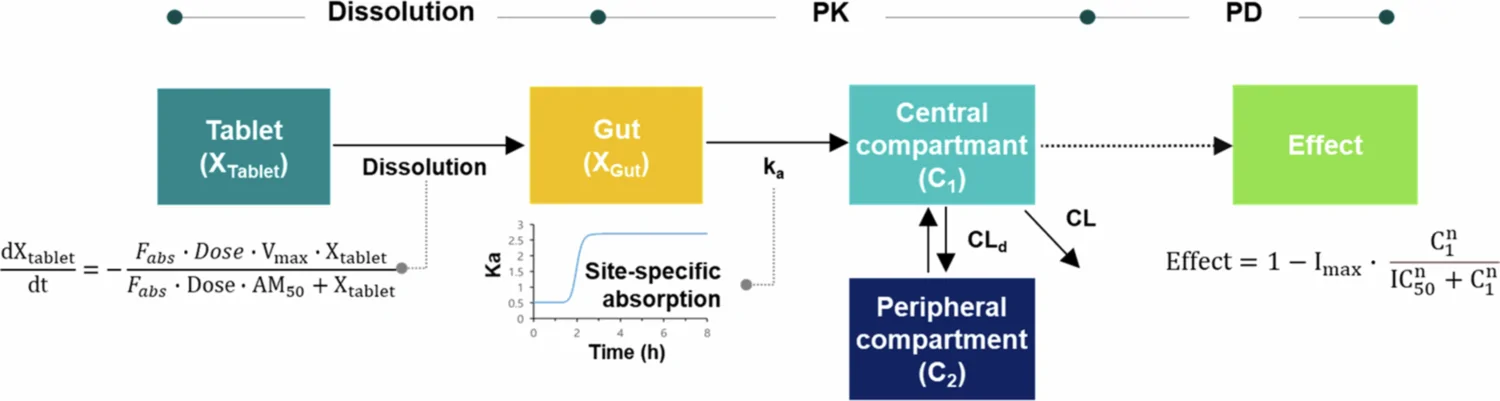
Schematic representation of the population pharmacokinetic/pharmacodynamic (PK/PD) model of ticagrelor SR tablets.

The population PK/PD parameter estimates are summarized in Table 5. The systemic clearance (CL) and volumes of distribution for the central (V_c_) and peripheral (V_p_) compartments were estimated at 12.6 L/h, 13.2 L, and 27.8 L, respectively. The estimated V_max, in vivo_ values for V1 (fast), V3 (medium), and V5 (slow) were 0.483, 0.230, and 0.178 h^−1^, respectively, reflecting the intended differences in dissolution rate among the three formulations. For the PD parameters, the I_max_ was estimated at 0.998, suggesting near-complete inhibition of platelet aggregation at sufficiently high concentrations, with an IC_50_ of 9.68 ng/mL and a Hill coefficient (*n*) of 1.91. The goodness-of-fit plots confirmed adequate agreement between the observed and predicted values for both PK and PD at the population and individual levels (Figure 6).

Some parameters showed higher standard errors (SE%), particularly the BSV estimates and a few population mean estimates (Table 5). The higher SE% may reflect the difficulty of estimating these parameters from limited data. However, most of the parameters with high SE% have very small point estimates, indicating that their contribution to the model output is limited. Sizeable BSV itself was also observed for distribution and absorption parameters, which may reflect the physiological heterogeneity across animals.

**Table 5.** Population pharmacokinetic and pharmacodynamic parameter estimates of ticagrelor by the PK/PD model.

Parameter	Symbol	Unit	Population mean (SE%)	BSV (SE%)
V_d_ of the central compartment	V_c_	L	13.2 (17.6%)	0.207 (131%)
V_d_ of the peripheral compartment	V_p_	L	27.8 (22.5%)	0.732 (147%)
Systemic clearance	CL	L/h	12.6 (18.9%)	0.17 (99.8%)
Distribution clearance	CL_d_	L/h	28 (19.1%)	0.507 (108%)
Amount ratio at half-maximum dissolution rate	AM_50_	-	0.0351 (46.5%)	0.236 (155%)
Maximum in vivo dissolution rate of V1 (fast)	V_max, fast_	h^−1^	0.483 (32%)	0.123 (216%)
Maximum in vivo dissolution rate of V3 (medium)	V_max, med_	h^−1^	0.23 (19.5%)	0.0337 (504%)
Maximum in vivo dissolution rate of V5 (slow)	V_max, slow_	h^−1^	0.178 (4.9%)	0.0399 (422%)
Lag time in dissolution in the gastrointestinal tract	T_lag_	h	0.0594 (105%)	0.415 (176%)
Basal absorption rate constant	k_a_’	h^−1^	0.365 (29.2%)	0.82 (60.3%)
Relative increment in the absorption rate constant at the site of absorption	Abs_max_	-	5.48 (8%)	0.122 (209%)
Time to reach the midpoint of absorption enhancement	T_get_	h	2.47 (10.5%)	0.329 (53%)
Hill coefficient for absorption	γ	-	18.1 (55.2%)	0.461 (338%)
Oral bioavailability	F_abs_	-	0.268 (29.2%)	0.232 (123%)
Maximum fractional inhibition	I_max_	-	0.998 (0.2%)	0.0001 (422%)
Concentration at half maximum inhibition	IC_50_	ng/mL	9.68 (3.4%)	0.00711 (718%)
Hill coefficient for inhibition	n	-	1.91 (6.6%)	0.0263 (307%)

Note: BSV, between-subject variability; SE, standard error.

**Figure 6.**
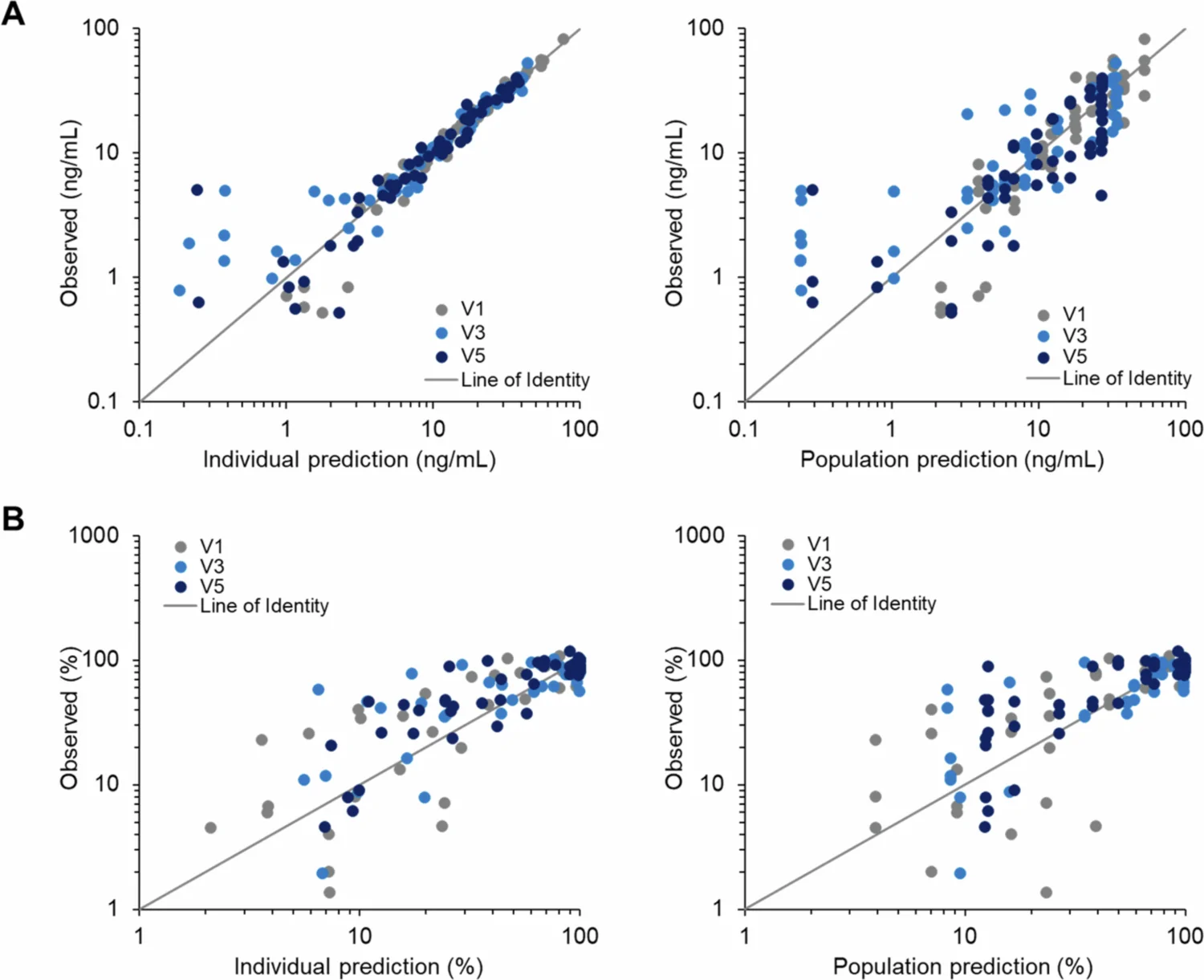
Goodness-of-fit plots for the population PK/PD model: plasma concentration (A) and platelet aggregation (B). Left panels show observed vs. individual-predicted values; right panels show observed vs. population-predicted values. The solid line represents the line of identity.

Model robustness and parameter identifiability were further assessed by bootstrap and local sensitivity analyzes. Of 200 bootstrap replicates, 86% converged successfully, and the bootstrap medians of most parameters agreed closely with the original estimates, with larger uncertainty confined to the parameters describing the absorption transition and to the variability components (Supplementary Table S2). In the sensitivity analysis, V_max, in vivo_ was the dominant driver of C_max_ and the PD responses among the dissolution and absorption parameters, with its impact increasing from V1 to V5. AUC was determined solely by CL and F_abs_, and the PD responses were most sensitive to I_max_ and IC_50_, as expected from the model structure (Supplementary Table S3).

### 3.3. In vitro–in vivo correlation

Based on the established population PK/PD model, the time-dependent absorption rate constant (k_a_) and the predicted in vivo dissolution and absorption curves are illustrated in Figure 7. The estimated T_get_ of 2.47 h represents the inflection point of the sigmoidal transition, at which k_a_ reached 50% of its maximum enhancement. The high Hill coefficient (*γ* = 18.1) indicated that this transition was abrupt rather than gradual (Figure 7A). The predicted in vivo dissolution and absorption profiles agreed with the rank order of the in vitro dissolution rates across the three formulations (Figure 7B).

**Figure 7.**
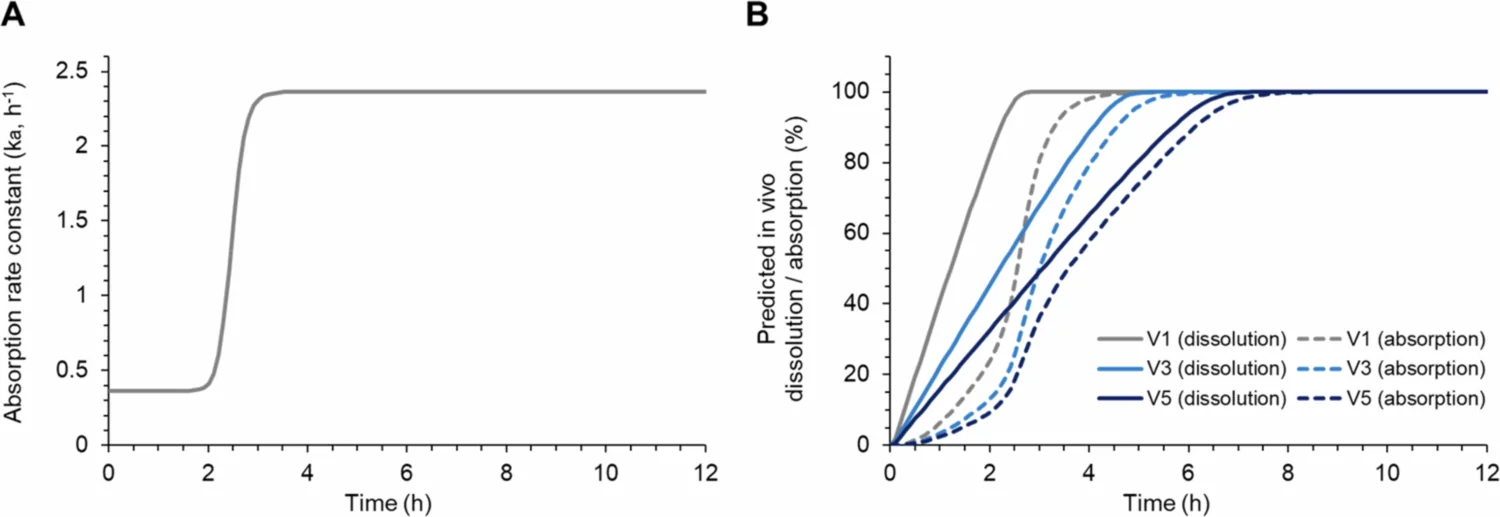
Time-dependent absorption rate constant (k_a_) profile (A) and predicted in vivo dissolution (solid lines) and absorption (dashed lines) profiles (B) for V1 (fast), V3 (medium), and V5 (slow), estimated by the population PK/PD model.

To establish an IVIVC, the estimated V_max, in vivo_ values were correlated with the corresponding V_max, in vitro_ values. The V_max, in vivo_ values were consistently higher than their V_max, in vitro_ counterparts (Figures 2B and 7B), indicating faster dissolution under physiological conditions. An exponential regression adequately described the relationship between V_max, in vivo_ and V_max, in vitro_ (Figure 8A):

(11) $$V_{\max,\;\mathrm{invivo}}=0.1178\;∙\;e^{3.6524\;∙\;V_{\max,\;\mathrm{invitro}}}$$

with an R^2^ of 0.994. Given the shared AM_50_ across formulations, V_max_ served as the sole formulation-specific determinant of the dissolution profile, and this correlation therefore captured the underlying profile-to-profile relationship in parameter form. Using Equation (11), the in vivo dissolution profiles were converted back to the in vitro scale, and the predicted in vitro dissolution was compared with the observed values point-to-point (Figure 8B). The close agreement along the line of identity confirmed the establishment of a Level A IVIVC for ticagrelor SR tablets, consistent with the point-to-point relationship definition in the FDA guidance (FDA US 1997).

**Figure 8.**
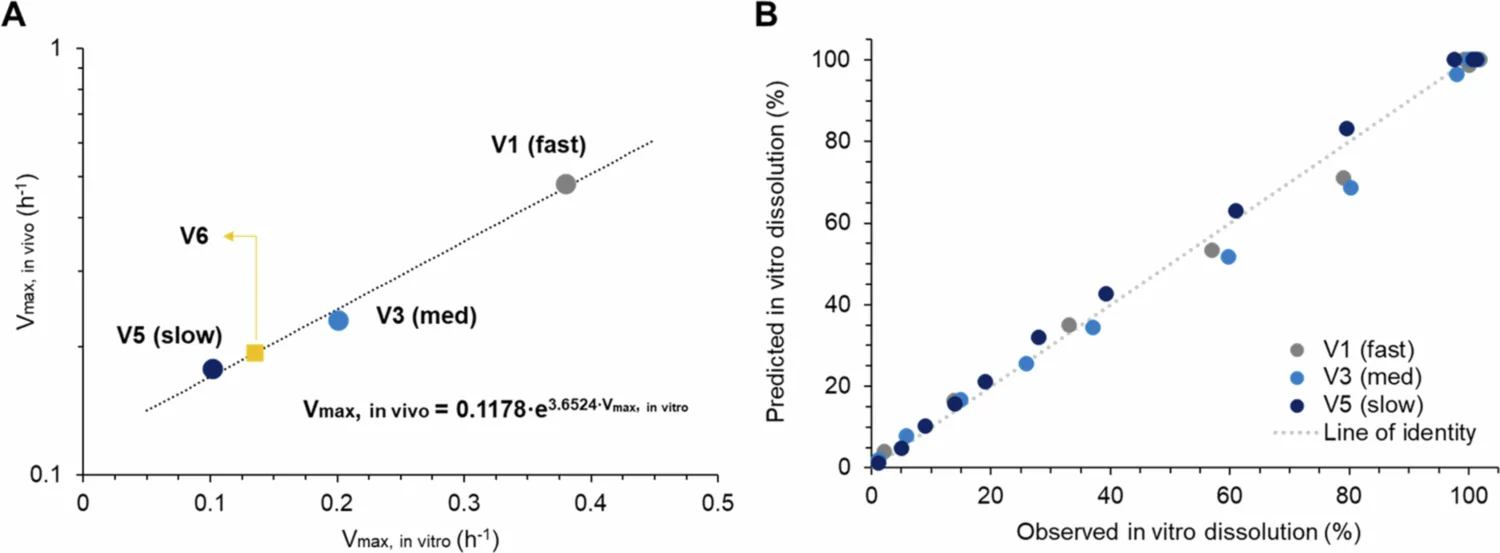
In vitro–in vivo correlation (IVIVC) of ticagrelor SR tablets. Correlation between V_max, in vitro_ and V_max, in vivo_ with exponential regression (R^2^ = 0.994) (A) and point-to-point correlation between the observed and predicted in vitro dissolution from in vivo data (B). V6 (yellow square) was not used in the regression.

### 3.4. Integrated framework

#### 3.4.1. Model development

The DoE equation (Equation (10)) and IVIVC equation (Equation (11)) were combined with the population PK/PD model to form the extended IVIVC–PK/PD framework. This integrated framework enables direct prediction of in vitro dissolution, in vivo dissolution, plasma concentration–time, and antiplatelet effect–time profiles from formulation composition. The framework's predictive performance was evaluated using validation formulations (Section 3.4.2).

For validation, the plasma concentration–time and platelet aggregation–time profiles of V1 (fast), V3 (medium), and V5 (slow) were simulated by Monte Carlo simulation (*n* = 1,000) and compared with the observed data. The visual predictive check (VPC) plots are presented in Figure 9. The majority of the observed data points fell within the 5^th^–95^th^ percentile prediction intervals for both PK (upper panels) and PD (lower panels), indicating that the model adequately captured the central tendency and variability of the observed profiles across all three formulations. The prediction intervals suggest that the parameter uncertainties translate to intervals of appropriate width rather than systematic bias in the typical profile.

**Figure 9.**
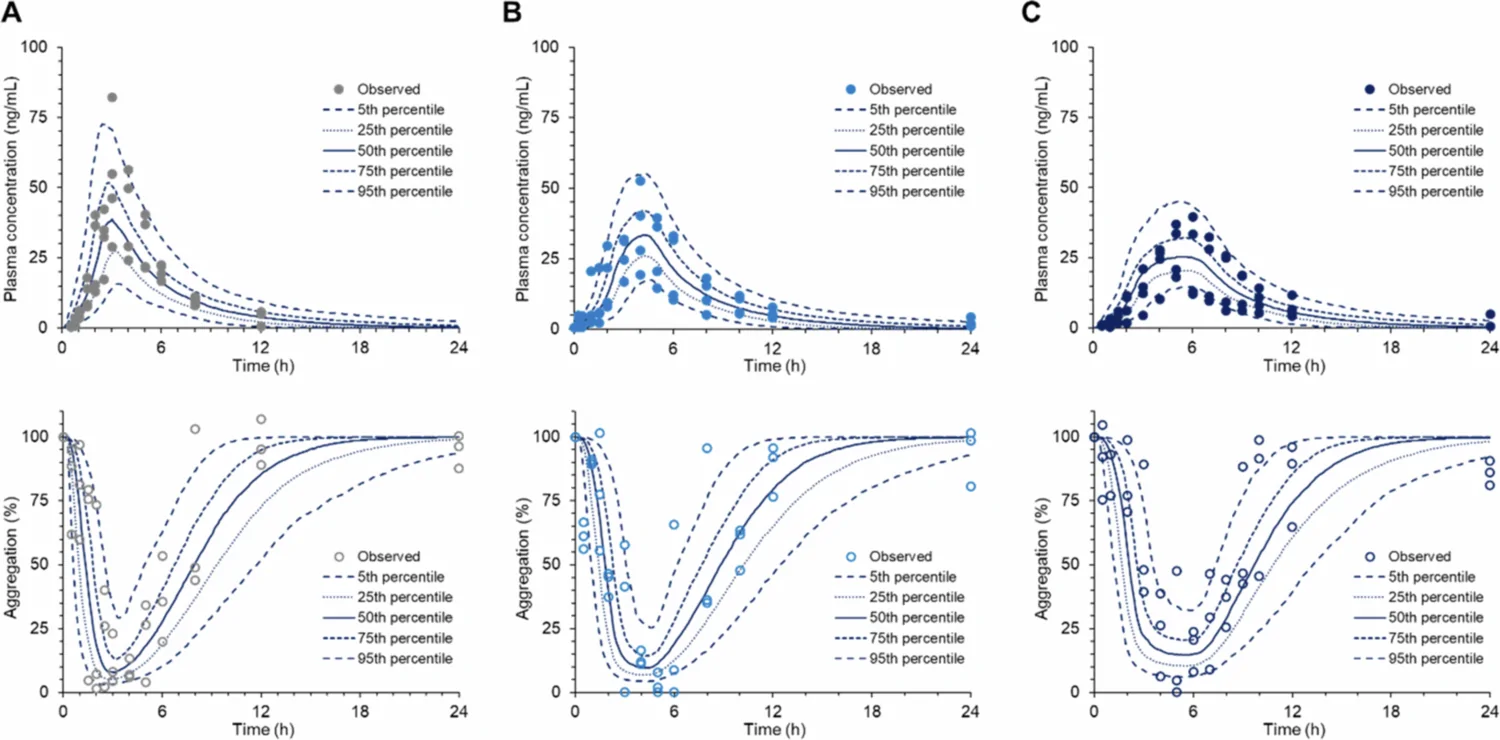
Visual predictive check (VPC) plots of the extended IVIVC model for V1 (fast) (A), V3 (medium) (B), and V5 (slow) (C). Upper panels: plasma concentration–time profiles; lower panels: platelet aggregation–time profiles. Solid lines represent the 50^th^ percentile; dashed and dotted lines represent the 5^th^, 25^th^, 75^th^, and 95^th^ percentiles. Symbols represent individual observed data.

Table 6 summarizes the predicted and observed PK and PD parameters. For the PK parameters, the PE% for C_max_ and AUC_last_ were less than 12.70% and 9.80%, respectively, with mean values of 7.65% and 4.93%. For the PD parameters, the PE% for E_max_ and AUEC were less than 7.53% and 11.96%, respectively, with mean values of 5.71% and 6.88%.

**Table 6.** Observed and predicted pharmacokinetic (PK) and pharmacodynamic (PD) parameters of ticagrelor SR tablets for validation (V1, V3, V5) and application (V6) of the extended IVIVC model.

Parameter			Validation			Application
			V1 (fast)	V3 (medium)	V5 (slow)	V6
PK	C_max_ (ng/mL)	Observed (*n* = 4)	56.26 ± 18.45	35.12 ± 14.54	29.67 ± 8.52	31.12 ± 13.80
		Predicted	49.12	37.63	28.75	31.87
		PE (%)	12.70	7.14	3.10	2.43
	AUC_last_ (ng·h/mL)	Observed (*n* = 4)	208.13 ± 27.41	240.31 ± 93.03	206.25 ± 116.68	193.45 ± 99.90
		Predicted	213.17	216.77	211.58	212.15
		PE (%)	2.42	9.80	2.58	9.67
PD	E_max_ (%)	Observed (*n* = 3)	96.71 ± 1.67	97.37 ± 4.56	90.58 ± 12.59	86.30 ± 17.29
		Predicted	93.00	90.04	85.37	86.83
		PE (%)	3.84	7.53	5.75	0.61
	AUEC (%·h)	Observed (*n* = 3)	630.09 ± 46.04	740.57 ± 14.16	722.34 ± 174.54	727.42 ± 144.84
		Predicted	705.45	746.16	779.57	764.92
		PE (%)	11.96	0.76	7.92	5.15

#### 3.4.2. Model application

To demonstrate the practical utility of the extended IVIVC-PK/PD model, a formulation composition (V6) that was not used in model development or validation was selected from the design space (Table 1, Figure 2A). V6 was designed to exhibit a dissolution rate between those of V3 (medium) and V5 (slow), as shown in the contour plot (Figure 2A) and the in vitro dissolution profiles (Figure 2B, Table 2). Using the formulation composition as the sole input, the in vivo PK and PD profiles of V6 were predicted via Monte Carlo simulation (*n* = 1,000). Separately, V6 tablets were prepared and administered to beagle dogs (*n* = 4) to obtain the observed PK and PD data. Figure 10 shows the VPC plots comparing the model-predicted and observed plasma concentration–time (A) and platelet aggregation–time (B) profiles of V6. The majority of the observed data points fell within the 5^th^–95^th^ percentile prediction intervals for both PK and PD, demonstrating that the extended IVIVC-PK/PD model adequately predicted the in vivo performance of an untested formulation. The PE% for pharmacokinetic endpoints (C_max_ and AUC_last_) were 2.43% and 9.67%, respectively, meeting the FDA criteria for IVIVC predictability (FDA US 1997). Comparable prediction accuracy was achieved for pharmacodynamic endpoints (E_max_ and AUEC: 0.61% and 5.15%, respectively; Table 6), for which no equivalent regulatory criterion currently exists.

**Figure 10.**
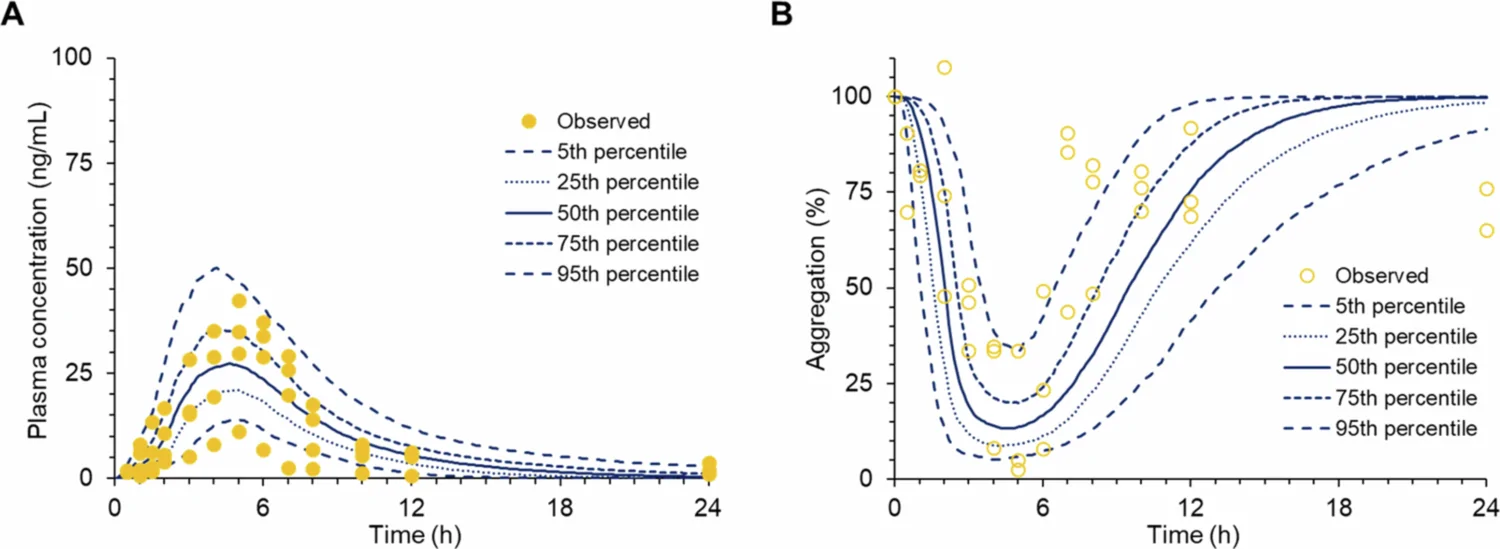
Application of the extended IVIVC model to V6. The plasma concentration–time (A) and platelet aggregation–time (B) profiles were predicted from formulation composition alone (lines) and compared with the observed data (symbols).

Using the validated framework, the effect of formulation composition on the PK and PD responses was also explored (Supplementary Figure S2). C_max_ varied with composition while AUC remained unchanged. The PD responses were considerably less sensitive because the inhibition was already close to its plateau (Table 6); AUEC remained largely comparable across compositions, decreasing slightly with increasing C_max_.

## 4. Discussion

The present study demonstrates that formulation composition can be directly translated into pharmacokinetic and pharmacodynamic outcomes through an extended IVIVC-PK/PD framework. Using ticagrelor as a model drug, nineteen SR formulations with different compositions were prepared by mixture design, and the DoE, population PK/PD model, and IVIVC were sequentially established from in vitro dissolution and in vivo PK/PD data obtained in beagle dogs. The framework could predict the plasma concentration–time and platelet aggregation–time profiles of an independent formulation (V6) solely from its formulation composition, with prediction errors for both PK and PD less than 10%. These prediction errors satisfied the FDA criteria for conventional IVIVC (FDA US 1997), despite the present framework extending the prediction range in both directions—from in vitro dissolution to formulation composition as an input, and from in vivo pharmacokinetics to pharmacodynamic response as an output.

The concept of extending IVIVC to formulation composition was first introduced for baclofen SR tablets, in which a single formulation factor, the HPMC content (%), was correlated with in vitro dissolution and in vivo pharmacokinetics (Kim et al. 2019). This approach was further advanced for ketoprofen SR tablets by incorporating a mixture design of DoE with multiple formulation factors (Lim et al. 2022). In both previous studies, however, the prediction endpoint has been limited to the plasma concentration–time profile. Beyond these two studies, formulation design frameworks that link composition to a pharmacodynamic response–time profile have rarely been attempted. The paucity likely reflects the practical and modeling constraints of extending formulation design to pharmacodynamic endpoints, which requires both measurable pharmacodynamic markers and extensive in vivo data across multiple formulations.

The present study extends the extended IVIVC framework to pharmacodynamic prediction by adding a pharmacodynamic modeling component to the framework. Ticagrelor was selected as the model drug because its antiplatelet effect is directly related to plasma concentration and can be quantified by whole blood impedance aggregometry. Once the integrated framework is established with a limited set of formulations, in vivo pharmacokinetic and pharmacodynamic profiles of additional formulations can be predicted directly from formulation composition without further in vivo testing, substantially reducing the extensive in vivo data requirement.

The extended IVIVC-PK/PD model was constructed by integrating three components (Figure 11). First, the mixture design of DoE established the mathematical relationship between formulation composition (Avicel, HPMC 15,000, and HPMC 100,000) and in vitro dissolution kinetics, with V_max, in vitro_ of the Michaelis-Menten equation serving as the response variable. Second, the population PK/PD model was developed to estimate in vivo dissolution parameters from the plasma concentration–time and platelet aggregation–time data, incorporating time-dependent changes in dissolution and absorption to account for gastrointestinal transit. Third, the IVIVC was established by correlating V_max, in vitro_ and V_max, in vivo_ by exponential regression. These three components were integrated via V_max_ as a common linking parameter: DoE converts formulation composition to V_max, in vitro_, IVIVC converts V_max, in vitro_ to V_max, in vivo_, and the population PK/PD model converts V_max, in vivo_ to plasma concentration and pharmacodynamic response. This integrated structure enables end-to-end prediction from formulation variables to pharmacokinetic as well as pharmacodynamic responses.

**Figure 11.**
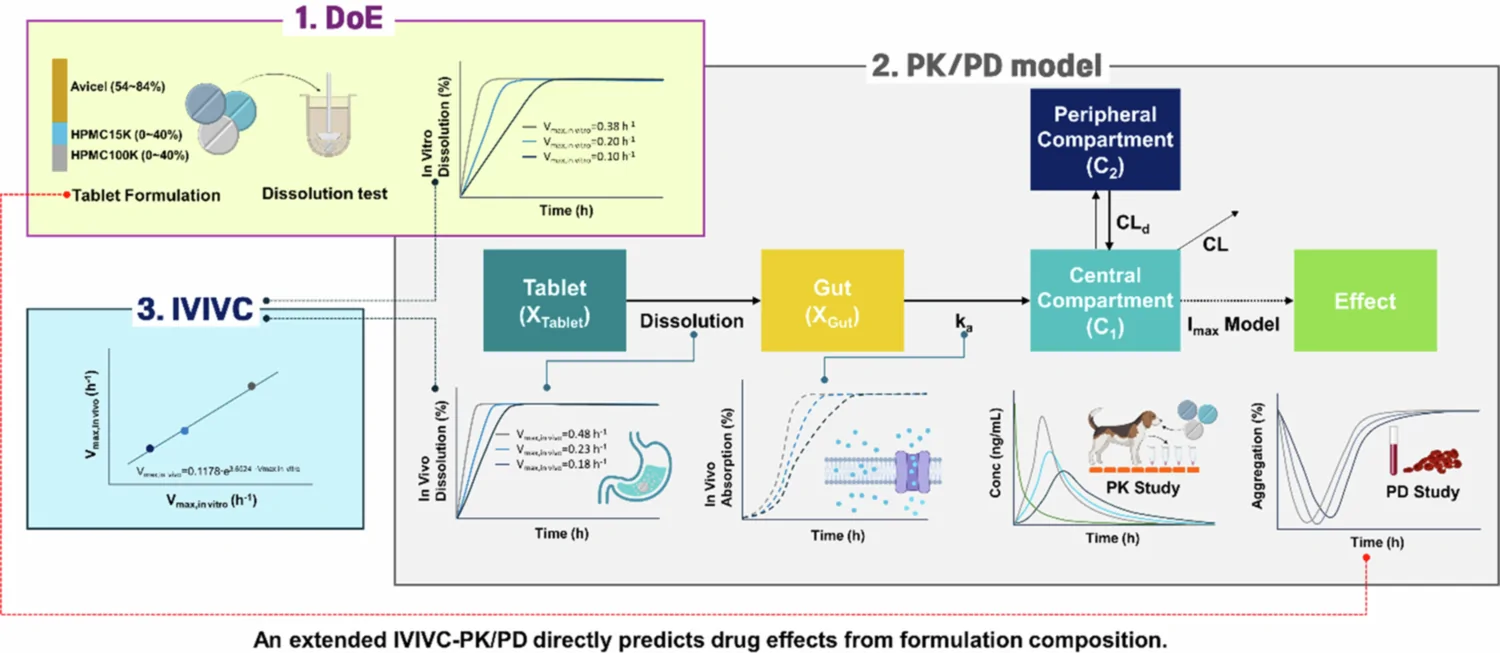
Schematic representation of the extended IVIVC–PK/PD framework comprising three integrated components: (Siepmann and Siepmann 2012) DoE converting formulation composition to V_max, in vitro_, (Mackie et al. 2024) a population PK/PD model converting V_max, in vivo_ to plasma concentration and pharmacodynamic response, and (FDA US 1997) IVIVC correlating V_max, in vitro_ with V_max, in vivo_, enabling direct prediction of both pharmacokinetic and pharmacodynamic responses from SR tablet formulation.

The mixture design identified that increasing the composition of HPMC 15,000 or HPMC 100,000 at the expense of Avicel resulted in slower dissolution (Figure 2), as expected from their less hydrophilic and more viscous nature. These in vitro differences translated directly into the in vivo pharmacokinetic profiles: V1 (fast-releasing), V3 (medium-releasing), and V5 (slow-releasing) showed progressively lower C_max_ and prolonged T_max_, while AUC_last_ remained comparable across the three formulations (Figure 3A, Table 3). These findings suggest that formulation-dependent release kinetics primarily modulated the rate, rather than the extent, of absorption. The slower terminal decline observed with slower-releasing formulations indicates flip-flop kinetics, where the terminal phase is governed by the absorption rate rather than the elimination rate. The comparable AUC_last_ values, despite different release rates, further confirm that the extent of absorption was preserved, while only the absorption rate was modulated by the formulation composition.

The population pharmacokinetic model incorporated a time-dependent absorption term to represent the physiological changes during gastrointestinal transit inferred from the plasma concentration profiles. The estimated parameters suggested that the fraction of drug available for absorption increased over time, consistent with the improved absorptive environment as the tablet moves from the stomach to the small intestine. The absorption enhancement began at approximately 2 h and reached its midpoint (T_get_) at 2.47 h (Figure 7A), consistent with the reported gastric emptying time in fasting beagle dogs (71–100 min) (Sagawa et al. 2009; Schmitz et al. 2010). Similar site-dependent absorption patterns have been reported for acyclovir (Shin et al. 2020). For ticagrelor, both dissolution and absorption appeared to be influenced by gastrointestinal transit, consistent with its BCS class IV characteristics.

The incorporation of pharmacodynamic assessment into SR formulation development represents a distinctive aspect of the present study. The time course of platelet aggregation clearly reflected the PK profiles, with the onset and recovery of antiplatelet effect corresponding to the rise and decline of plasma ticagrelor concentrations (Figure 3). All three SR formulations produced near-maximum inhibition of platelet aggregation, and AUEC values were comparable across formulations (Table 4). Although the AUEC of V1 appeared numerically smaller, this may be because the fast-releasing formulation achieved near-complete inhibition, beyond which further inhibitory effect cannot be captured. Nevertheless, the population PK/PD model adequately captured the time course of platelet aggregation for all three formulations, and the extended IVIVC-PK/PD model could predict the PD profiles of both the validation (V1, V3, V5) and application (V6) formulations from their formulation composition.

The antiplatelet effect of ticagrelor has been extensively characterized in clinical studies. The exposure–response relationship for platelet inhibition in patients with stable coronary artery disease and prior myocardial infarction has been characterized using a sigmoidal E_max_ model with direct effect (Astrand et al. 2019). The PK/PD model structure employed in the present study is consistent with these clinical findings. The extended IVIVC-PK/PD framework is designed to be transferable: once PK/PD model parameters are re-estimated using human data, the DoE and IVIVC components can be retained to predict clinical outcomes from formulation composition. An important feature of the present framework is that it does not simply predict pharmacodynamic outcomes from formulation composition. Instead, the model mechanistically connects formulation composition to a cascade of intermediate processes, including in vivo dissolution, in vivo absorption dynamics, and plasma concentration–time profiles, which ultimately determine pharmacodynamic response. As a result, the framework enables the simultaneous prediction of multiple levels of in vivo behavior—from formulation-dependent dissolution and absorption to systemic exposure and pharmacological effect. This hierarchical linkage allows formulation variables to be quantitatively connected to pharmacodynamic responses, supporting rational formulation design.

Taken together, the extended IVIVC-PK/PD model enables the prediction of both PK and PD profiles directly from formulation composition, thereby reducing the need for iterative in vitro and in vivo studies during formulation optimization. Furthermore, the model can be applied in reverse to identify formulation compositions that achieve a target pharmacodynamic profile. While the framework was evaluated using a single drug and formulation type in an animal model, its structure allows extension to other compounds and controlled-release systems with appropriate model adaptation. Validation with human clinical data will be important to confirm the translational utility of this framework.

Several limitations should be considered in interpreting the present framework. The framework was developed with a limited number of animals per group (PK, *n* = 4; PD, *n* = 3), and evaluated with a single drug, a single SR formulation platform, and a beagle dog model. Accordingly, the between-subject variability components were estimated with less precision than the population mean parameters, as reflected in the bootstrap analysis. While external validation with V6 supported the framework's predictive performance, broader validation with additional drugs, formulations, and species is required to establish generalizability. The model also involves simplifying assumptions in the time-dependent absorption and direct-inhibition PD representations, which may become insufficient under chronic dosing or in other physiological contexts. Translation to human pharmacokinetic and pharmacodynamic responses would additionally require accounting for interspecies differences in gastrointestinal physiology, drug metabolism, and platelet function, and verification with human data. With such validation and extension, the framework structure could support formulation design decisions across drugs, formulations, and eventually human applications.

## 5. Conclusions

An integrated IVIVC–PK/PD framework was developed and validated for ticagrelor sustained-release formulations, linking formulation composition to pharmacokinetic and pharmacodynamic responses. By enabling formulation design to be guided by pharmacological response rather than empirical iteration, the framework allows target pharmacodynamic profiles to be translated into formulation composition. This approach provides a practical and mechanistically informed strategy for rational, outcome-driven development of sustained-release formulations, supporting a shift from empirical formulation optimization toward model-informed design. Broader applicability to other drugs, formulations, species, and human studies will require further validation.

## Supplementary Material

Supplementary Material ARRIVE_Author Checklist_Full_submitted.pdf

Supplementary Material Supplementary material.docx

## Data Availability

All data generated or analyzed during this study are included in this published article and supplementary material.

## AI-assisted writing

Claude (Anthropic, Claude Opus 4.7) was used for language editing and paraphrasing during manuscript preparation. The authors reviewed all AI-assisted text and take full responsibility for the content.
