# Supplementary material for: Linking formulation composition to pharmacokinetic and pharmacodynamic responses: a predictive framework for sustained-release tablets
Source: Drug Deliv. 2026 Oct 27;33(1):2752652. doi: 10.1080/10717544.2026.2752652 (PMC13715338; doi:10.1080/10717544.2026.2752652)
Supplement: Supplementary file 2 [file IDRD_A_2752652_SM8774.docx]

**Supplementary Figure S1.** Contour plots showing the effects of the formulation composition on the in vitro dissolution parameters: (A) time to 80% dissolution (T_80_) and (B) reciprocal of maximum dissolution rate (1/V_max, in vitro_). The trapezoidal boundary represents the design space.

**Supplementary Figure S2.** Effect of formulation composition on predicted (A) C_max_, (B) AUC, (C) E_max_, and (D) AUEC. Values are % of the V3 reference for perturbations of Avicel (x_1_: −15%, −5%, +10%, +25%, limited by its feasible range), HPMC 15,000 (x_2_), and HPMC 100,000 (x_3_; ±25% and ±50%).

**Supplementary Table S1.** ANOVA of the responses of the experimental design for ticagrelor SR tablets.

| Response | Source | DF | Sequential sum of squares | Adjusted mean of squares | F value | P value |
| --- | --- | --- | --- | --- | --- | --- |
| T_80_ | Model | 8 | 148.482 | 18.5602 | 886.11 | 0 |
|  | Linear | 2 | 129.931 | 2.8896 | 137.96 | 0 |
|  | x_1_x_2_ | 1 | 0.06 | 1.9644 | 93.78 | 0.001 |
|  | x_1_x_3_ | 1 | 10.305 | 4.8421 | 231.17 | 0 |
|  | x_1_x_2_x_3_ | 1 | 3.509 | 4.1771 | 199.42 | 0 |
|  | x_1_x_2_(x_1_-x_2_) | 1 | 0.029 | 1.921 | 91.71 | 0.001 |
|  | x_1_x_3_(x_1_-x_3_) | 1 | 4.334 | 3.775 | 180.23 | 0 |
|  | x_2_x_3_(x_2_-x_3_) | 1 | 0.314 | 0.3135 | 14.97 | 0.018 |
|  | Residual | 4 | 0.084 | 0.0209 |  |  |
|  | Cor total | 12 | 148.566 |  |  |  |
| 1/V_max_ | Model | 8 | 196.51 | 24.5637 | 442.37 | 0 |
|  | Linear | 2 | 171.211 | 3.8386 | 69.13 | 0.001 |
|  | x_1_x_2_ | 1 | 0.007 | 2.34 | 42.14 | 0.003 |
|  | x_1_x_3_ | 1 | 11.62 | 6.4831 | 116.76 | 0 |
|  | x_1_x_2_x_3_ | 1 | 6.306 | 5.7132 | 102.89 | 0.001 |
|  | x_1_x_2_(x_1_-x_2_) | 1 | 0.191 | 2.4067 | 43.34 | 0.003 |
|  | x_1_x_3_(x_1_-x_3_) | 1 | 6.237 | 5.1808 | 93.3 | 0.001 |
|  | x_2_x_3_(x_2_-x_3_) | 1 | 0.937 | 0.9371 | 16.88 | 0.015 |
|  | Residual | 4 | 0.222 | 0.0555 |  |  |
|  | Cor total | 12 | 196.732 |  |  |  |

**Supplementary Table S2.** Bootstrap estimates of the population PK/PD model parameters.

| Parameter | Population mean | | | BSV | | |
| --- | --- | --- | --- | --- | --- | --- |
|  | Model estimate | Bootstrap | | Model estimate | Bootstrap | |
|  |  | Median | 95% CI |  | Median | 95% CI |
| V_c_ (L) | 13.219 | 12.792 | 10.234 – 16.848 | 0.207 | 0.185 | 0.015 – 0.272 |
| V_p_ (L) | 27.850 | 28.274 | 18.854 – 44.161 | 0.732 | 0.799 | 0.019 – 1.324 |
| CL (L/h) | 12.638 | 12.808 | 9.931 – 15.584 | 0.170 | 0.142 | 0.016 – 0.240 |
| CL_d_ (L/h) | 28.020 | 27.187 | 15.681 – 38.307 | 0.507 | 0.356 | 0.038 – 0.734 |
| AM_50_ | 0.035 | 0.016 | 0.004 – 0.075 | 0.236 | 0.255 | 0.115 – 0.662 |
| V_max, fast_ (h^-1^) | 0.483 | 0.458 | 0.383 – 0.586 | 0.123 | 0.073 | 0.023 – 0.147 |
| V_max, med_ (h^-1^) | 0.230 | 0.247 | 0.220 – 1.510 | 0.034 | 0.027 | 0.014 – 0.940 |
| V_max, slow_ (h^-1^) | 0.178 | 0.182 | 0.154 – 0.195 | 0.040 | 0.023 | 0.007 – 0.072 |
| T_lag_ (h) | 0.059 | 0.073 | 0.027 – 0.128 | 0.415 | 0.192 | 0.057 – 0.502 |
| k_a_’ (h^-1^) | 0.365 | 0.276 | 0.182 – 0.557 | 0.820 | 0.202 | 0.029 – 0.904 |
| Abs_max_ | 5.478 | 4.812 | 2.521 – 13.634 | 0.122 | 0.123 | 0.050 – 1.017 |
| T_get_ (h) | 2.471 | 2.525 | 1.906 – 3.359 | 0.329 | 0.307 | 0.109 – 0.453 |
| γ | 18.069 | 15.871 | 7.261 – 74.774 | 0.461 | 0.449 | 0.124 – 1.528 |
| F_abs_ | 0.268 | 0.277 | 0.187 – 0.373 | 0.232 | 0.249 | 0.021 – 0.373 |
| I_max_ | 0.998 | 0.998 | 0.995 – 0.999 | 0.001 | 0.001 | 0.000 – 0.003 |
| IC_50_ (ng/mL) | 9.683 | 9.771 | 9.551 – 9.847 | 0.007 | 0.007 | 0.004 – 0.016 |
| n | 1.908 | 1.875 | 1.852 – 1.917 | 0.026 | 0.013 | 0.009 – 0.021 |

Values are medians and 95% confidence intervals from 200 bootstrap replicates. BSV, between-subject variability; CI, confidence interval.

**Supplementary Table S3.** Local sensitivity analysis of the population PK/PD model parameters for V1, V3, and V5. Values are C_max_, AUC, E_max_, and AUEC as % of reference (Ref = 100), listed in the order −50% / −25% / +25% / +50% parameter perturbation.

| Form | Parameter | C_max_ | AUC | E_max_ | AUEC |
| --- | --- | --- | --- | --- | --- |
| V1 | V_max, fast_ | 63.9 / 91.4 / 96.4 / 93.7 | 100 / 100 / 100 / 100 | 95.4 / 99.3 / 99.7 / 99.5 | 104.4 / 100.1 / 100.8 / 101.3 |
|  | k_a_' | 80.3 / 92.7 / 104.3 / 106.9 | 100 / 100 / 100 / 100 | 98.2 / 99.4 / 100.3 / 100.4 | 101.9 / 100.4 / 99.9 / 99.8 |
|  | Abs_max_ | 81.2 / 91.8 / 106.6 / 112.1 | 100 / 100 / 100 / 100 | 98.3 / 99.4 / 100.4 / 100.7 | 103.2 / 101.2 / 99.2 / 98.7 |
|  | T_get_ | 87 / 101.1 / 88.3 / 77.6 | 100 / 100 / 100 / 100 | 98.9 / 100.1 / 99 / 97.8 | 96.5 / 97.2 / 103.4 / 106.6 |
|  | γ | 92.5 / 97.1 / 101.9 / 103.2 | 100 / 100 / 100 / 100 | 99.4 / 99.8 / 100.1 / 100.2 | 100.1 / 100 / 100 / 100 |
|  | I_max_ | 100 / 100 / 100 / 100 | 100 / 100 / 100 / 100 | 50 / 75 / 125 / 150 | 50 / 75 / 125 / 150 |
|  | IC_50_ | 100 / 100 / 100 / 100 | 100 / 100 / 100 / 100 | 102.7 / 101.5 / 98.1 / 96 | 143.1 / 117.8 / 86.5 / 75.8 |
|  | n | 100 / 100 / 100 / 100 | 100 / 100 / 100 / 100 | 86.9 / 95.6 / 102.1 / 103 | 111.1 / 102.8 / 99 / 98.6 |
|  | V_c_ | 117.7 / 108.2 / 93 / 86.9 | 100 / 100 / 100 / 100 | 101 / 100.5 / 99.5 / 98.9 | 95.7 / 97.9 / 102 / 104 |
|  | V_p_ | 113.9 / 105.3 / 96.4 / 93.8 | 100 / 100 / 100 / 100 | 100.8 / 100.3 / 99.7 / 99.5 | 86.9 / 94.4 / 104.1 / 107 |
|  | CL | 118.5 / 108.4 / 92.8 / 86.6 | 199.7 / 133.3 / 80 / 66.7 | 101 / 100.5 / 99.5 / 98.9 | 188.9 / 129.9 / 81.8 / 69.5 |
|  | CL_d_ | 116 / 106.5 / 95.4 / 91.9 | 100 / 100 / 100 / 100 | 100.9 / 100.4 / 99.7 / 99.4 | 96.3 / 99.2 / 100.2 / 100.2 |
|  | F_abs_ | 50 / 75 / 125 / 150 | 50 / 75 / 125 / 150 | 91 / 97.4 / 101.3 / 102 | 60 / 82.7 / 113.8 / 125.1 |
|  | AM_50_ | 99.7 / 99.9 / 99.9 / 99.6 | 100 / 100 / 100 / 100 | 100 / 100 / 100 / 100 | 100 / 100 / 100 / 100 |
|  | T_lag_ | 99.6 / 99.8 / 100.2 / 100.4 | 100 / 100 / 100 / 100 | 100 / 100 / 100 / 100 | 100.2 / 100.1 / 99.9 / 99.8 |
| V3 | V_max, med_ | 56 / 75.9 / 122.4 / 140 | 100 / 100 / 100 / 100 | 87.4 / 95.3 / 102.3 / 103.5 | 117.6 / 105.5 / 97.6 / 96.8 |
|  | k_a_' | 92.2 / 96.4 / 102.2 / 103.2 | 100 / 100 / 100 / 100 | 98.8 / 99.5 / 100.3 / 100.4 | 101.5 / 100.1 / 100.2 / 100.5 |
|  | Abs_max_ | 91.1 / 95.1 / 104.3 / 108 | 100 / 100 / 100 / 100 | 98.6 / 99.3 / 100.6 / 101 | 103 / 101.1 / 99.3 / 98.8 |
|  | T_get_ | 86.7 / 89.3 / 111.1 / 118 | 100 / 100 / 100 / 100 | 97.8 / 98.3 / 101.3 / 102 | 102.6 / 100.7 / 100.5 / 102 |
|  | γ | 96.4 / 98.6 / 100.9 / 101.4 | 100 / 100 / 100 / 100 | 99.5 / 99.8 / 100.1 / 100.2 | 100.7 / 100.2 / 99.9 / 99.8 |
|  | I_max_ | 100 / 100 / 100 / 100 | 100 / 100 / 100 / 100 | 50 / 75 / 125 / 150 | 50 / 75 / 125 / 150 |
|  | IC_50_ | 100 / 100 / 100 / 100 | 100 / 100 / 100 / 100 | 105.5 / 103.1 / 96.3 / 92.3 | 143.1 / 117.7 / 86.6 / 76.1 |
|  | n | 100 / 100 / 100 / 100 | 100 / 100 / 100 / 100 | 84.3 / 94 / 103.5 / 105.4 | 111 / 102.8 / 98.9 / 98.3 |
|  | V_c_ | 113.8 / 106.4 / 94.5 / 89.9 | 100 / 100 / 100 / 100 | 101.6 / 100.8 / 99.2 / 98.4 | 96.1 / 98.1 / 101.9 / 103.7 |
|  | V_p_ | 118.4 / 106.7 / 95.7 / 92.8 | 100 / 100 / 100 / 100 | 102 / 100.8 / 99.4 / 98.9 | 88.1 / 94.9 / 103.8 / 106.5 |
|  | CL | 126.1 / 109.8 / 92 / 85.4 | 199.6 / 133.3 / 80 / 66.7 | 102.6 / 101.2 / 98.8 / 97.5 | 186.1 / 128.9 / 82.4 / 70.5 |
|  | CL_d_ | 116.4 / 106.4 / 95.8 / 94.4 | 100 / 100 / 100 / 100 | 101.8 / 100.8 / 99.4 / 99.2 | 97 / 99.3 / 100.2 / 100.1 |
|  | F_abs_ | 50 / 75 / 125 / 150 | 50 / 75 / 125 / 150 | 83.6 / 95 / 102.5 / 104 | 60.2 / 82.8 / 113.7 / 125.1 |
|  | AM_50_ | 103 / 101.5 / 98.6 / 97.3 | 100 / 100 / 100 / 100 | 100.4 / 100.2 / 99.8 / 99.6 | 99.3 / 99.6 / 100.4 / 100.7 |
|  | T_lag_ | 100.7 / 100.3 / 99.7 / 99.3 | 100 / 100 / 100 / 100 | 100.1 / 100 / 100 / 99.9 | 100 / 100 / 100 / 100 |
| V5 | V_max, slow_ | 58.1 / 80.7 / 122.7 / 145.7 | 100 / 100 / 100 / 100 | 81.8 / 94.2 / 104.1 / 106.7 | 121 / 107.9 / 95.4 / 92.8 |
|  | k_a_' | 99.5 / 100.1 / 100.9 / 101.8 | 100 / 100 / 100 / 100 | 99.9 / 100 / 100.2 / 100.4 | 101.2 / 100.1 / 100.2 / 100.5 |
|  | Abs_max_ | 98.5 / 99.6 / 103 / 106.5 | 100 / 100 / 100 / 100 | 99.6 / 99.9 / 100.7 / 101.4 | 102.2 / 100.8 / 99.5 / 99.1 |
|  | T_get_ | 96.1 / 97.6 / 111 / 120.8 | 100 / 100 / 100 / 100 | 99 / 99.4 / 102.3 / 103.8 | 103.7 / 101.6 / 99.2 / 99 |
|  | γ | 99.9 / 100 / 100 / 100.1 | 100 / 100 / 100 / 100 | 100 / 100 / 100 / 100 | 100.6 / 100.2 / 99.9 / 99.8 |
|  | I_max_ | 100 / 100 / 100 / 100 | 100 / 100 / 100 / 100 | 50 / 75 / 125 / 150 | 50 / 75 / 125 / 150 |
|  | IC_50_ | 100 / 100 / 100 / 100 | 100 / 100 / 100 / 100 | 109.8 / 105.4 / 93.9 / 87.5 | 142 / 117.4 / 86.6 / 75.8 |
|  | n | 100 / 100 / 100 / 100 | 100 / 100 / 100 / 100 | 83 / 92.8 / 105 / 108.3 | 109.3 / 102 / 99.5 / 99.5 |
|  | V_c_ | 112.2 / 105.1 / 97.6 / 95.2 | 100 / 100 / 100 / 100 | 102.5 / 101.1 / 99.4 / 98.8 | 96.8 / 98.4 / 101.5 / 103 |
|  | V_p_ | 119.1 / 108.3 / 94.6 / 91.6 | 100 / 100 / 100 / 100 | 103.6 / 101.8 / 98.7 / 97.8 | 90.4 / 95.8 / 103.1 / 105.4 |
|  | CL | 145.6 / 118.9 / 90.9 / 84.3 | 199.6 / 133.3 / 80 / 66.7 | 106.7 / 103.6 / 97.6 / 95.5 | 180.5 / 127.1 / 83.4 / 72.1 |
|  | CL_d_ | 115.1 / 105.2 / 100.8 / 101.5 | 100 / 100 / 100 / 100 | 103 / 101.1 / 100.2 / 100.3 | 97.9 / 99.5 / 100.1 / 100.1 |
|  | F_abs_ | 50 / 75 / 125 / 150 | 50 / 75 / 125 / 150 | 74.8 / 91.8 / 104.4 / 107 | 59.3 / 82.8 / 113.5 / 124.6 |
|  | AM_50_ | 104.5 / 102.1 / 98.2 / 96.6 | 100 / 100 / 100 / 100 | 101 / 100.5 / 99.6 / 99.2 | 99 / 99.5 / 100.5 / 101 |
|  | T_lag_ | 100.1 / 100.1 / 99.9 / 99.9 | 100 / 100 / 100 / 100 | 100 / 100 / 100 / 100 | 99.9 / 100 / 100 / 100.1 |
